# Comprehensive characterization of endoplasmic reticulum stress in bladder cancer revealing the association with tumor immune microenvironment and prognosis

**DOI:** 10.3389/fgene.2023.1097179

**Published:** 2023-04-07

**Authors:** Yuda Lin, Tengfei Li, Zhuolun Li, Chong Shen, Zhouliang Wu, Zhe Zhang, Zhi Li, Shaobo Yang, Zejin Wang, Peng Li, Chong Fu, Jian Guo, Hailong Hu

**Affiliations:** ^1^ Department of Urology, The Second Hospital of Tianjin Medical University, Tianjin, China; ^2^ Tianjin Key Laboratory of Urology, Tianjin Institute of Urology, The Second Hospital of Tianjin Medical University, Tianjin, China; ^3^ Tianjin Children’s Hospital, Tianjin, China

**Keywords:** bladder cancer, endoplasmic reticulum stress, molecular subtype, tumor microenvironment, prognostic

## Abstract

**Background:** This study constructs a molecular subtype and prognostic model of bladder cancer (BLCA) through endoplasmic reticulum stress (ERS) related genes, thus helping to clinically guide accurate treatment and prognostic assessment.

**Methods:** The Bladder Cancer (BLCA) gene expression data was downloaded from The Cancer Genome Atlas (TCGA) and Gene Expression Omnibus (GEO) database. We clustered by ERS-related genes which obtained through GeneCards database, results in the establishment of a new molecular typing of bladder cancer. Further, we explored the characteristics of each typology in terms of immune microenvironment, mutations, and drug screening. By analyzing the ERS-related genes with univariate Cox, LASSO and multivariate Cox analyses, we also developed the four-gene signature, while validating the prognostic effect of the model in GSE32894 and GSE13507 cohorts. Finally, we evaluated the prognostic value of the clinical data in the high and low ERS score groups and constructed a prognostic score line graph by Nomogram.

**Results:** We constructed four molecular subtypes (C1- C4) of bladder cancer, in which patients with C2 had a poor prognosis and those with C3 had a better prognosis. The C2 had a high degree of TP53 mutation, significant immune cell infiltration and high immune score. In contrast, C3 had a high degree of FGFR3 mutation, insignificant immune cell infiltration, and reduced immune checkpoint expression. After that, we built ERS-related risk signature to calculate ERS score, including ATP2A3, STIM2, VWF and P4HB. In the GSE32894 and GSE13507, the signature also had good predictive value for prognosis. In addition, ERS scores were shown to correlate well with various clinical features. Finally, we correlated the ERS clusters and ERS score. Patients with high ERS score were more likely to have the C2 phenotype, while patients with low ERS score were C3.

**Conclusion:** In summary, we identified four novel molecular subtypes of BLCA by ERS-related genes which could provide some new insights into precision medicine. Prognostic models constructed from ERS-related genes can be used to predict clinical outcomes. Our study contributes to the study of personalized treatment and mechanisms of BLCA.

## Introduction

Bladder cancer (BLCA) is the fourth most common cancer in male and the 11th most common cancer in female, with extremely high rates of recurrence and progression (Siegel et al., 2022). In recent years, treatment strategies for bladder cancer are not only traditional surgery and chemotherapy, but also immunotherapy (Abd El-Salam et al., 2022). Although immunotherapy has led to benefits for some patients, there are still patients with advanced bladder cancer who have had less benefit from immunotherapy (Afonso et al., 2020). Therefore, more immunotherapy targets still need to be developed to benefit patients with bladder cancer.

Endoplasmic reticulum stress is an attempt by cells to prevent the accumulation of misfolded or unfolded proteins, thereby activating the unfolded protein response (UPR) (So, 2018). ERS is identified by protein inositol-acquiring enzyme 1 (IRE1), protein kinase R (PKR)-like ER kinase (PERK), and activating transcription factor 6 (ATF6) that reside in the ER. Tumor cells are exposed to factors that alter protein homeostasis over time, resulting in ERS (Urra et al., 2016). Studies have shown that the tumor microenvironment (TME) can induce ERS and activate immune responses (Matsuo et al., 2013; Salvagno et al., 2022). In addition, ERS is also expected to be a new target for drug research in the treatment of tumors (Liu et al., 2022). However, studies in bladder cancer are still relatively rare, including promising studies of ERS in the microenvironment and treatment of bladder cancer.

This study comprehensively investigated the importance of ERS-related genes in BLCA. Novel molecular subtypes and prognostic signature of BLCA were constructed to innovatively explore the underlying mechanisms of ERS and bladder cancer. In addition, this study also correlated ERS with TME, gene mutation, and precision therapy in BLCA.

## Materials and methods

### ERS-related genes and database sources

No ethnical approval nor informed consent was required in this study to the public availability of data in the public database. Based on the GeneCards database, 258 ERS-related genes were obtained (Supplementary Tables S1, S2), and the screening criteria were protein-coding genes with a correlation score greater than 12. Obtain BLCA gene expression matrix, clinical information and mutation information through the TCGA website (Blum et al., 2018). GSE cohorts were gathered from Gene Expression Omnibus (GEO), namely, GSE32894 and GSE13507 (Barrett et al., 2013). The training dataset was TCGA-BLCA, accepting samples from 400 patients with survival information. 224 samples of GSE32894 cohort and 165 samples of GSE13507 cohort with valid survival time was used for validation (Kim et al., 2010; Sjödahl et al., 2012). Obtain the expression matrix and patient information for the IMvigor210 cohort *via* the “IMvigor210CoreBiologies” R package (Necchi et al., 2017). TISCH database was used to characterize the expression of the signature genes in different kinds of cells of bladder cancer tissue (Sun et al., 2021).

### Function and pathway enrichment analyses

To investigate the pathways associated with ERS-related genes in BLCA, we used the “clusterProfiler” package in R to perform GO and KEGG enrichment analysis on ERS related genes (Yu et al., 2012) and the pathways obtained from MsigDB website (Liberzon et al., 2015). ERS-related pathways (Supplementary Table S3), tumor-related pathways (Supplementary Table S4) and metabolism-related pathway were also available from the MsigDB website (https://www.gsea-msigdb.org/gsea/msigdb/). In addition, to explore the mechanisms of each molecular subtyping more precisely, we collected stromal-related pathways from a study conducted by Mariathasan et al. (2018) (Supplementary Table S5). The “GSVA” package was used to calculate the enrichment score for TCGA samples (Hänzelmann et al., 2013).

### Cluster Analysis

We performed consensus clustering analysis of ERS-related genes using the “ConsensusClusterPlus” package. The same clustering method was used for both the training and validation sets (Wilkerson and Hayes, 2010). GSE32894 cohort used for independent validation of our molecular subtypes.

### Mutational analysis

The “maftools” package was used for analysis of mutations in BLCA subtypes (Mayakonda et al., 2018). The top ten genes with the highest degree of mutation in each subtype are shown by waterfall diagram. The “TCGAmutations” package was used for obtaining TMB scores of TCGA patients.

### Depicting TME of BLCA

We used the “estimate” package for calculating immune, stromal and estimate scores. By using the TIMER method, immune infiltration analysis was performed on each sample of the four subtypes, thus comparing the immune microenvironment of each subtype (Li et al., 2017). To make the results more reliable, we still used CIBERSORT, CIBERSORT-ABS, QUANTISEQ, MCPCOUNTER, XCELL and EPIC methods to compare the immune infiltration of each subtype (Becht et al., 2016; Aran et al., 2017; Finotello et al., 2019; Racle and Gfeller, 2020; Le et al., 2021). Finally, we included the cancer immunity cycle (Chen and Mellman, 2013). The cycle reflects the 7 steps in which immune cells exert their anti-tumor effects (Supplementary Table S6).

### Molecular subtypes of BLCA

In order to achieve precise treatment of patients with bladder malignancies, many experts have already performed molecular subtypes of BLCA. There are seven main molecular typing methods (Consensus, TCGA, MDA, Lund, CIT, UNC, Baylor) (Sjödahl et al., 2012; Choi et al., 2014; Damrauer et al., 2014; Rebouissou et al., 2014; Robertson et al., 2017; Mo et al., 2018; Kamoun et al., 2020)that are currently recognized. “ConsensusMIBC” and “BLCAsubtyping” R packages were used to divided samples of TCGA and GSE32894 to different subtypes in our study. Although these different methods could classify samples into a variety of types, the vast majority of patients can be categorized as luminal and basal subtypes. Additionally, we collected 12 specific classical signatures of BLCA. These results were summarized in a figure in the form of a heat map.

### Chemotherapy and immunotherapy sensitivity analysis

We used a public database, Genomics of Drug Sensitivity in Cancer (https://www.cancerrxgene.org/), to assess the response of four subtypes to chemotherapy with four drugs (Yang et al., 2013). The drug response was estimated by calculating the half-maximal inhibitory concentration (IC50). Additionally, the tumour immune dysfunction and exclusion (TIDE) algorithm (Jiang et al., 2018) and Immunophenoscore (IPS) were used for predicting the sensitivity of immunotherapy (Charoentong et al., 2017).

### Construction and validation of the signature

First, through univariate cox analysis and “survival” R package, we identified prognosis-related genes from 258 ERS-related genes. Then, to avoid overfitting of the model, Lasso regression analysis was performed on prognosis-related genes. Genes with non-zero coefficients at the best lambda value were included in multivariable Cox analysis. Finally, we obtain the prognostic risk score signature for ERS-associated genes. The following is the prognostic scoring formula:

ERS score=Ʃβi*RNAi,where βi is the coefficient of the i−th.

After that, we divided the patients into high ERS score and low ERS score groups according to their median ERS scores. The Kaplan-Meier method was applied to plot the survival curves, while the log-rank test was applied to calculate statistical significance. The feasibility and accuracy of ERS score for predicting 1-, 3-, and 5-year outcomes were further assessed by generating ROC curves. Meanwhile, the ERS scores were also highly effective in two independent validation sets GSE32894 and GSE13507.

### Constructing comprehensive nomogram

For individualized assessment, we included stage, age, gender and ERS scores of the sample in univariate and multivariant Cox analysis. After that, we divided the age by 70 years into younger and older. The relationship between ERS score and age, gender, stage, survival, T-stage, N-stage and M-stage was further evaluated. We also evaluated the clinical outcomes of different types of patients in the high and low ERS score groups. Finally, stage, age and ERS score were used to construct comprehensive nomogram to enable prognostic assessment of individualized patients. In addition, we explored the correlation between the ERS score, the four molecular subtypes, and stage.

### Statistical analysis

The data were analyzed through the use of R software (version 4.1.3). We analyzed the correlations between variables using Pearson or Spearman coefficients. Statistical tests were two-sided, and the level of significance was set at *p* < 0.05.

### RNA sequencing of BLCA samples

An RNeasy Mini Kit (Qiagen, Valencia, CA) was used to extract total RNA of specimens. According to the manufacturer’s instructions RNA-seq was performed using the QuantSeq kit FWD HT kit (Lexogen) using 500 ng input RNA. We used NEBNext UltraTM RNA Library Prep Kit for Illumina (NEB, USA) to generate Sequencing libraries. Consequently, 125- to 150-bp paired-end reads were generated using an Illumina HiSeq platform. Additionally, 6 μg of total RNA per sample was used as the input material for the small RNA library using the NEBNext Multiplex Small RNA Library Prep Set for Illumina (NEB, Ipswich, MA). 50-bp single-end sequencing was performed on an Illumina Hiseq 4000 platform. HISAT2 aligner was used to align the raw reads to the human reference genome GRCh38. Finally, we obtained the baseline phase RNA sequencing results for 29 patients (named the TMU-BLCA cohort). All patients included in this study signed an informed consent form and were approved by the Ethics Committee of the Second Hospital of Tianjin Medical University. Clinical information for patients can be found at Supplementary Table S7.

### Cell culture

The BCa cell lines T24, UMUC3 and EJ were purchased at the Chinese Academy of Sciences Cell bank, and the 253J-BV was presented by Professor Li Lei of the First Affiliated Hospital of Xi’an Jiaotong University. All cells were cultured in RPMI1640 or MEM with 10% fetal bovine serum (FBS) at 37°C in a 5% CO2/95% air incubator.

### RNA extraction and real-time quantitative PCR (RT-qPCR)

For RT-qPCR, total RNA was extracted from 20 paired samples of bladder cancer and adjacent normal tissues by surgical excision in the Second Hospital of Tianjin Medical University. The specific situation of the patients is reflected in Supplementary Table S8. The total RNA isolation and subsequent RT-qPCR were conducted as previously described (Li et al., 2022). The primers sequences (Sangon Biotech) were as follow: ATP2A3: F CAT​CCT​GAC​GGG​TGA​ATC​TGT R TGC​CCG​ATG​TGA​TAT​TGG​TGC. STIM2: F AGA​CAA​CAA​TGT​CAA​AGG​AAC​GA R ACT​CCG​GTC​ACT​GAT​TTT​CAA​C. VWF: F CCG​ATG​CAG​CCT​TTT​CGG​A R TCC​CCA​AGA​TAC​ACG​GAG​AGG. P4HB: F GGC​TAT​CCC​ACC​ATC​AAG​TTC R TCA​CGA​TGT​CAT​CAG​CCT​CTC. GAPDH: F CGG​AGT​CAA​CGG​ATT​TGG​TC R TTC​CCG​TTC​TCA​GCC​TTG​AC.

## Results

### Function and pathway enrichment analyses

**FIGURE 1:**
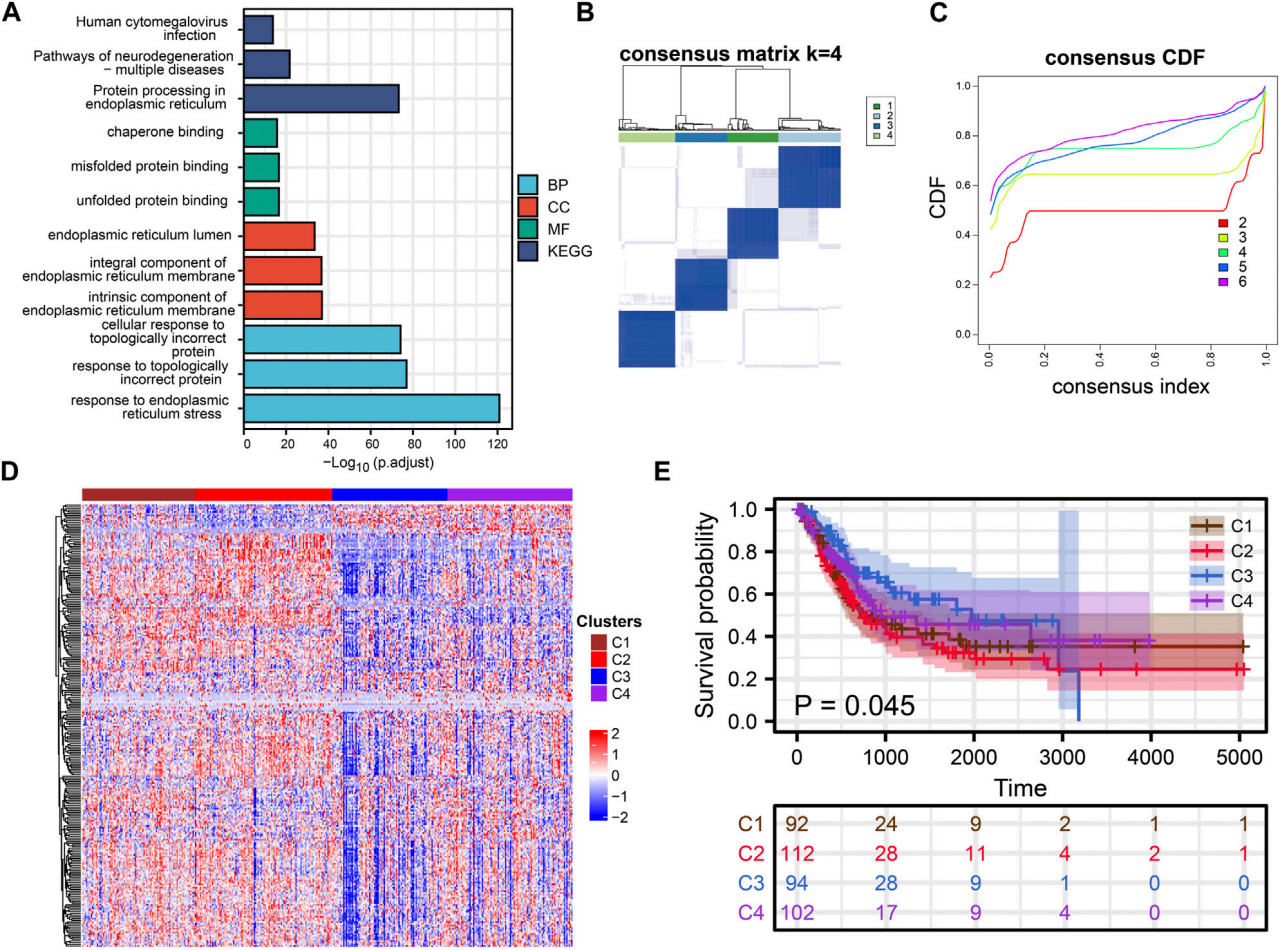
Cluster Analysis. **(A)** Rectangular diagram showing the results of GO and KEGG enrichment analysis. **(B, C)** Consensus clustering analysis of ERS related genes. **(D)** The heat map shows the expression of ERS-related genes in the four subtypes. **(E)** Kaplan-Meier curves demonstrate differences in clinical outcomes for the four subtypes.

ERS-related genes are mainly enriched in endoplasmic reticulum function-related GO and KEGG terms. In addition to this, they are also enriched in Human cytomegalovirus infection, Pathways of neurodegeneration-multiple diseases and response to topologically incorrect protein (Figure 1A). Overall, ERS related genes included in the study were significantly associated with endoplasmic reticulum stress, and they were also significantly enriched in the immune pathway. It confirmed a non-negligible relationship between ERS and the immune microenvironment.

### Cluster Analysis

Consensus clustering analysis of ERS genes classified BLCA into four subtypes (Figures 1B, C). These ERS-related genes were significantly different among the four subtypes, and more highly expressed genes were present in C1 and C2 (Figure 1D). Kaplan-Meier survival curves indicated that the C2 had the worst prognostic outcome, and the C3 had the best prognostic outcome (Figure 1E). With the same clustering approach, we observed the same phenomenon in the validation queue (Figures 3A–C). Combined with the clinical data, the C3 had younger age, better N-stage, pathological stage and T-stage (Supplementary Figures S1A–D). Similarly, the same phenomenon is observed in the verification cohort (Supplementary Figure S2). Thus, C3 may still be in the early stages of tumor development and patients have a better prognosis.

### Exploring the TME of 4 ERS subtypes

**FIGURE 2:**
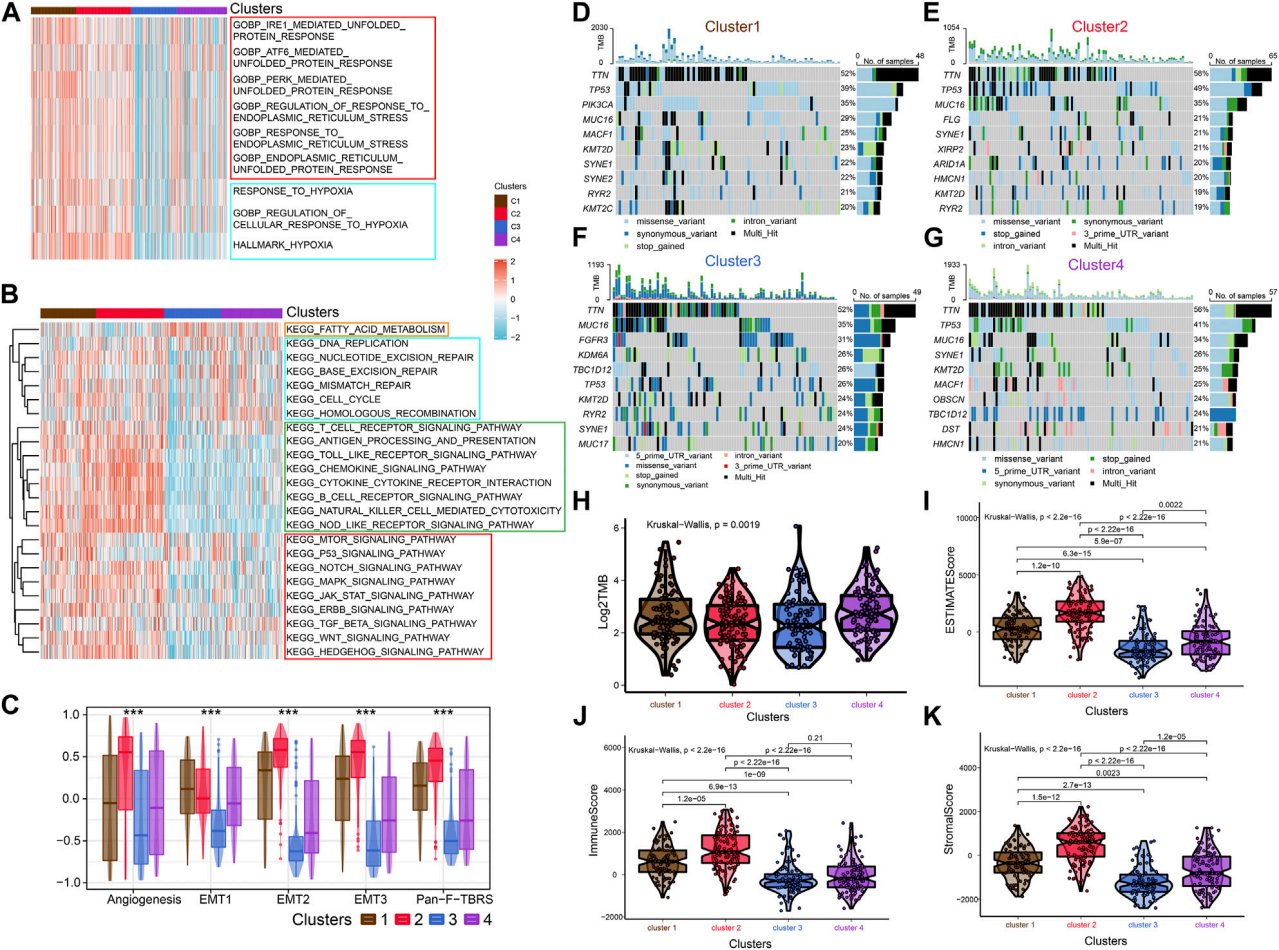
Enrichment analysis, Mutational analysis and Immune scoring. **(A)** The heat map shows the ERS-related pathways for the four subtypes. **(B)** The heat map shows the four subtype-related pathways. **(C)** Differences in Angiogenesis, EMT1, EMT2, EMT3 and Pan-F-TBRs scores across the four subtypes. **(D–G)** Waterfall diagram showing the top ten genes with high tumor mutational load for the four subtypes. **(H)** Differences in TMB scores across the four subtypes. **(I–K)** Comparison of estimate scores, immune scores and stromal scores in four subtypes.

To further investigate the characteristics of ERS in the four subtypes, we enriched the ERS-related pathways (Figure 2A). The results showed that ERS activity was significantly elevated in C1 and C2. Hypoxia affects ERO1α-mediated protein post-translational folding and disulfide bond formation leading to ERS (Chen and Cubillos-Ruiz, 2021). Interestingly, the activity of hypoxia-related pathways was also significantly elevated in C1 and C2. Similarly, we enriched tumor-associated pathways to explored the characteristics of the four isoforms precisely (Figure 2B). C3 was mainly associated with fatty acid metabolism, while C2 was mainly associated with immune pathways. Tumor-specific pathways such as MTOR signaling pathway, p53 signaling pathway as well as ERBB signaling pathway were enriched in C1, while others (NOTCH signaling pathway, MAPK signaling pathway, JAK-STAT signaling pathway, TGF-β signaling pathway, WNT signaling pathway and Hedgehog signaling pathway) were enriched in C2. C4 was associated with cell cycle and DNA damage repair related pathways. In addition, C2 had higher Angiogenesis, EMT1, EMT2, EMT3, and Pan-F-TBRs scores which means a higher stromal level may contribute to its worse prognosis (Figure 2C). We also explored metabolism-related pathways. The results showed that most metabolism related pathways were enriched in C1 and C2, while pyruvate metabolism, fatty acid metabolism, and tyrosine metabolism related pathways were enriched in C3 subgroup. It implied that differences in the metabolic microenvironment of different subtypes may influence to some extent the clinical outcome of patients (Supplementary Figure S2E). Mutational analysis revealed that unlike C3, which exhibited high tumor mutation burden in FGFR3, KDM6A, and TBC1D12, C2 exhibited high tumor mutation burden in TP53 and FLG (Figures 2D–G). The C4 has the highest total tumor mutation load (TMB) (Figure 2H). There are previous studies that reported FGFR3 mutations are strongly associated with earlier stage and longer survival time (van Rhijn et al., 2020). While TP53 mutations have opposite outcomes (Sjödahl et al., 2020). These researches coincided with our findings.

After that, we explored TME of C1-C4. C2 had a highest estimate score, immune score and stromal score while C3 was lowest both in TCGA and GSE32894 cohorts (Figures 2I–K–KFigures 2I–K, 3D). Depending on seven immune infiltration methods of analysis, C2 had more immune infiltration which followed by C1 subtype. Conversely, C3 has least immune infiltration (Figure 4A). Immunomodulators are important for assessing the immune microenvironment of tumors. The gene expression of immunomodulators was elevated mainly in C1 and C2, further validating the high immune activation status of C1 and C2, especially in C2 (Figure 4B). In addition, C2 had stronger cancer immunity cycle activity and there was more activation of anti-tumor immune cells (Figure 5A). The C2 immune checkpoint expression was significantly higher (Figure 5B). These results suggested that the C2 may have a poor clinical outcome due to significant tumor immune escape though with a stronger immune response. The C2 phenotype was also closely associated with high M2 macrophage infiltration (Figure 5C). There was substantial evidence that M2 macrophage polarization suppresses the immune microenvironment and causes a poorer outcome (Komohara et al., 2016; Wei et al., 2022). This further substantiated our perspective that C2 phenotype is associated with immune evasion. Further validation was also obtained in the validation queue (Supplementary Figures S2A, B, S3A, B).

**FIGURE 3:**
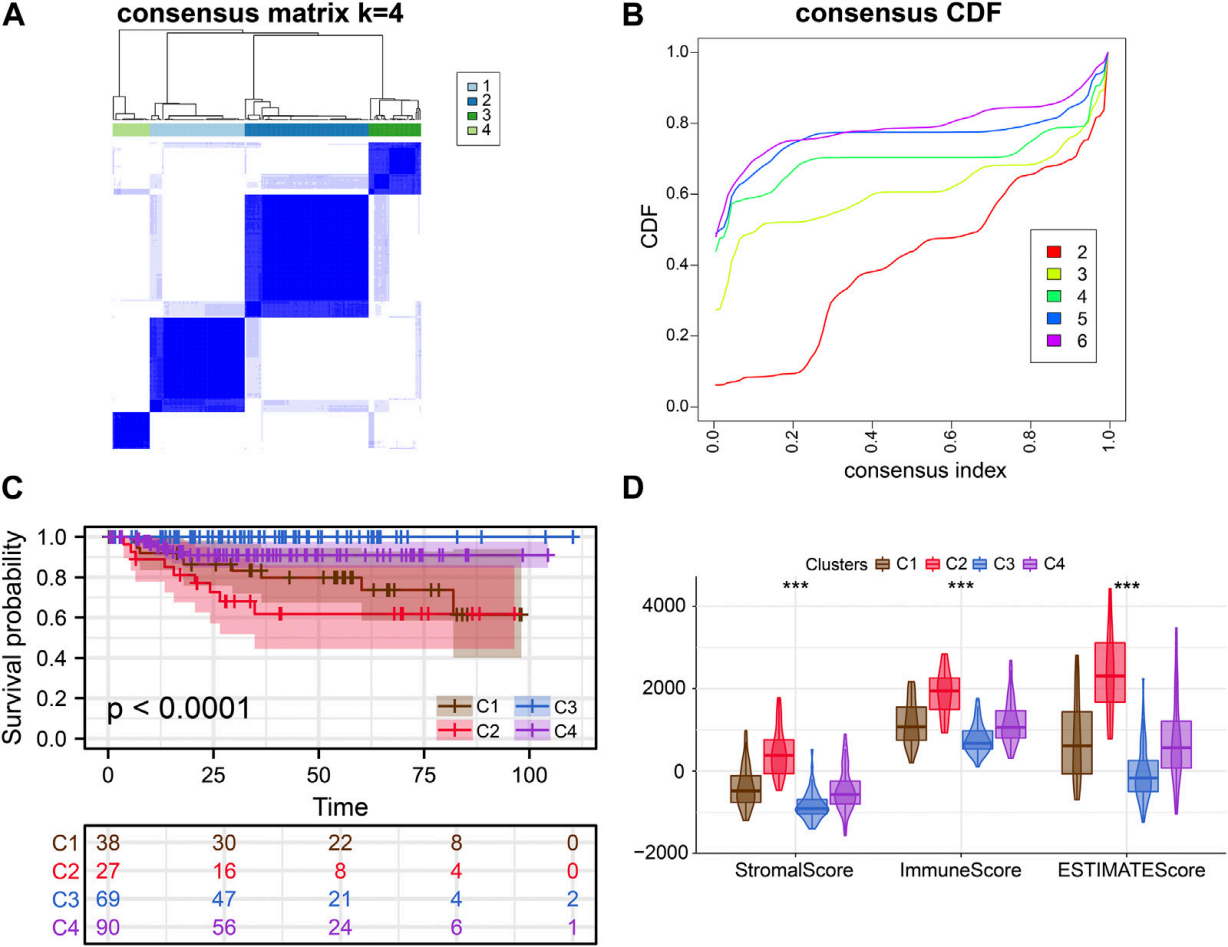
External verification of clusters. **(A, B)** Consensus clustering analysis of ERS-related genes. **(C)** Kaplan-Meier curves demonstrate differences in clinical outcomes for the four subtypes. **(D)** Comparison of estimate scores, immune scores and stromal scores in four subtypes.

**FIGURE 4:**
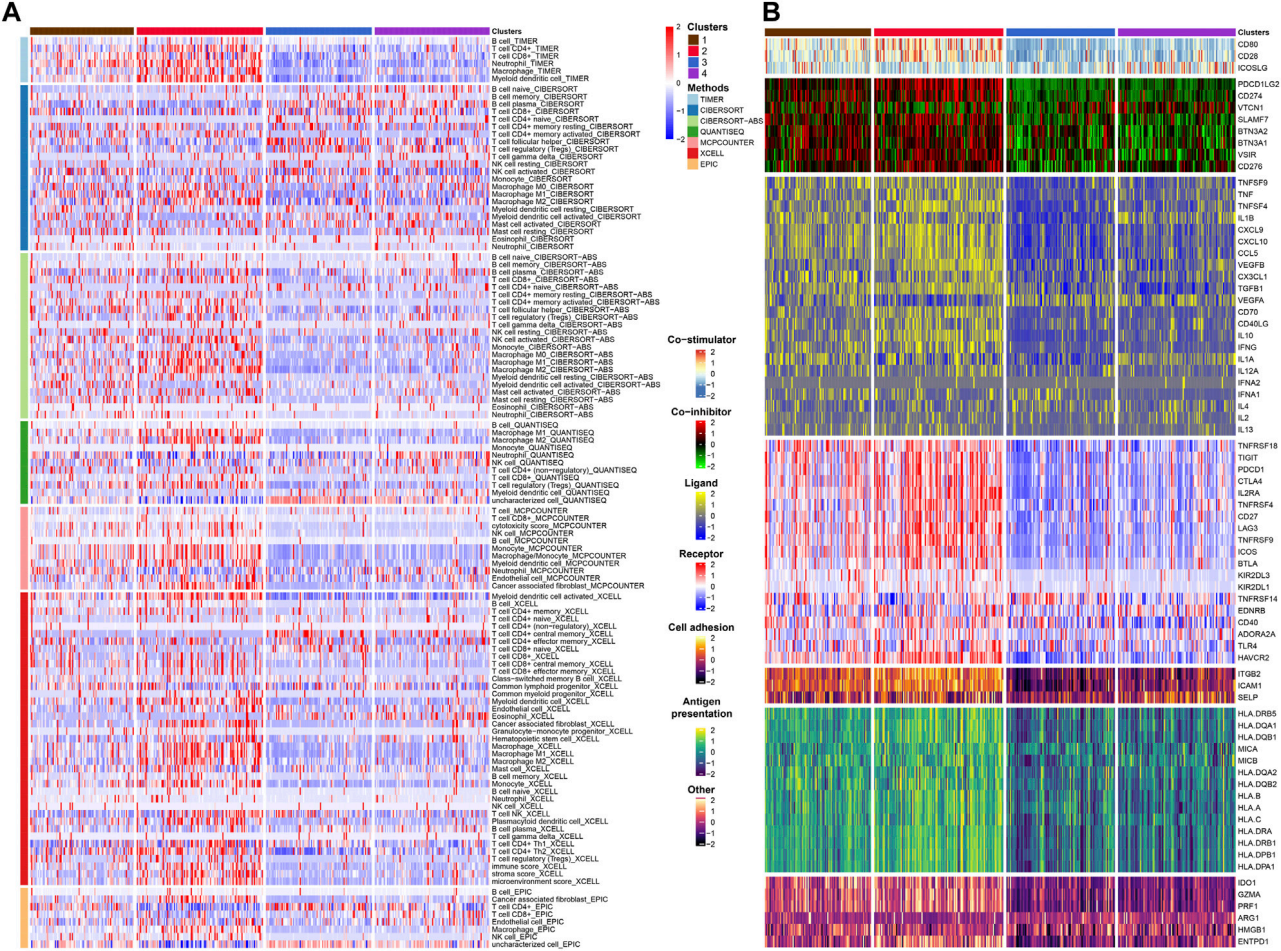
Immune infiltration and immunomodulators analysis. **(A)** Heat map showing the results of 7 methods to assess immune infiltration. **(B)** Heat map showing the differential expression of immunomodulator genes in the four subtypes.

**FIGURE 5:**
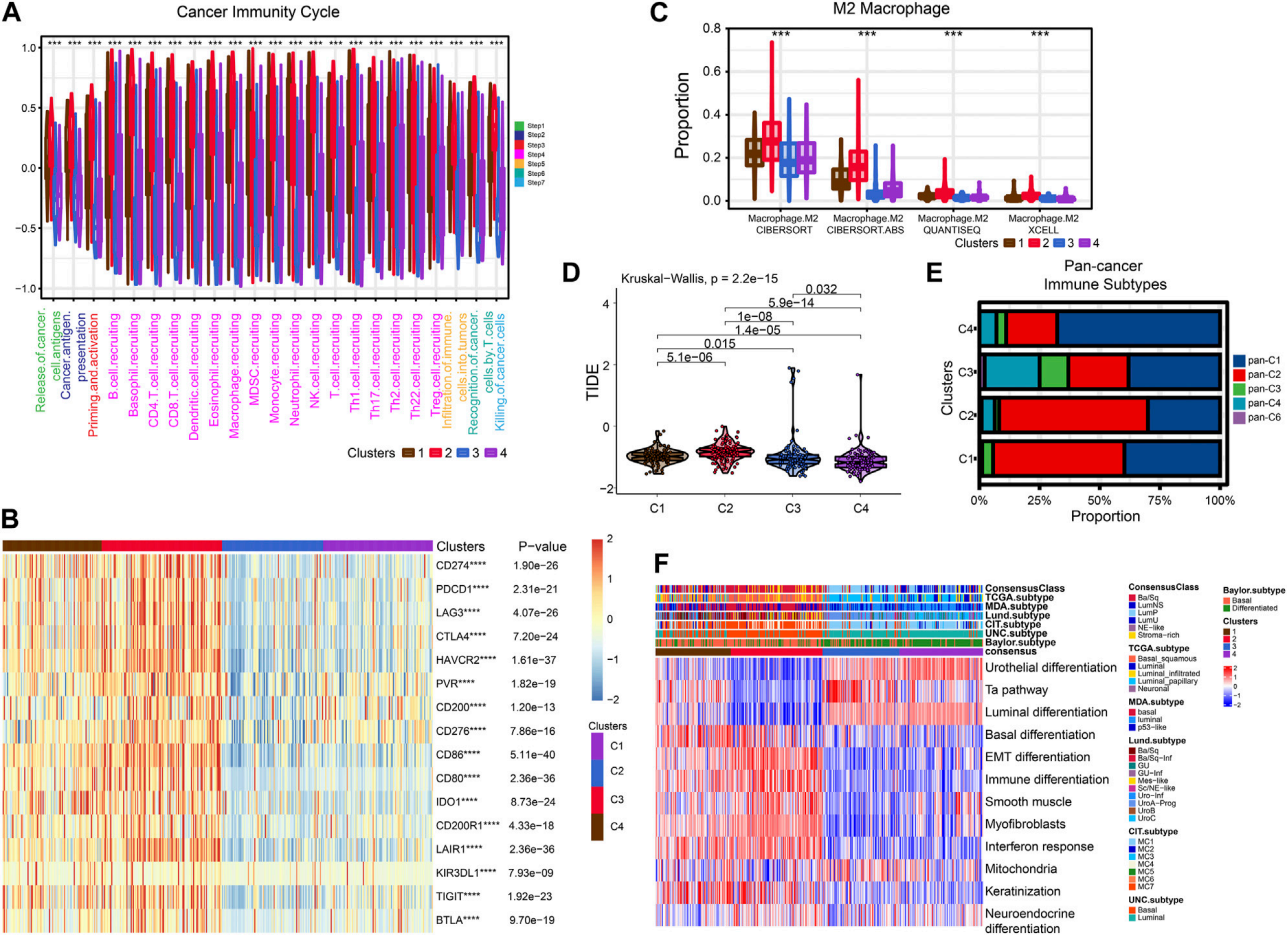
Exploration of tumor immune microenvironmen. **(A)** Four subtypes of cancer immunity cycle. **(B)** Heat map showing the difference in immune checkpoint expression between the four typologies. **(C)** Differential enrichment of M2 macrophages in four subtypes. **(D)** Violin plots showing the TIDE scores of different clusters. **(E)** Comparison of four subtypes and pan-cancer immune subtypes. **(F)** Comparison of four subtypes with BLCA subtypes.

Next, we compared these four subtypes with pan-cancer immune subtypes and BLCA molecular subtypes. We obtained the pan-cancer subtypes of TCGA BLCA samples from a previous study (Thorsson et al., 2018). The results showed that C1 and C2 were significantly associated with pan-C2 (IFN-γ) subtype, exhibiting the greatest amount of immune infiltration and poor prognosis, which is consistent with our study. The C3 was clearly associated with pan-C4 (Lymphocyte Depleted) subtype and shows lymphocyte depletion (Figure 5E). In comparison with the currently accepted molecular typing of bladder cancer, the C2 was more of the basal type, the C3 and C4 was more luminal subtype, and the C1 might be intermediate (Figure 5F). These results were similar for the validation cohort (Supplementary Figure S2C).

### Drug Sensitivity analysis

Cisplatin, Gemcitabine, Paclitaxel and Doxorubicin are the most common chemotherapy drugs for BLCA. All four of these drugs showed the best efficacy in C1 and C2 (Supplementary Figures S4A–D). Therefore, the C1 and C2 subtype may have better benefit with chemotherapy drugs. Higher TIDE scores indicate that patients will benefit more from immunotherapy. Thus, we speculation C4 may more suitable for immunotherapy (Figure 5D).

### Construction of prognostic signature

**FIGURE 6:**
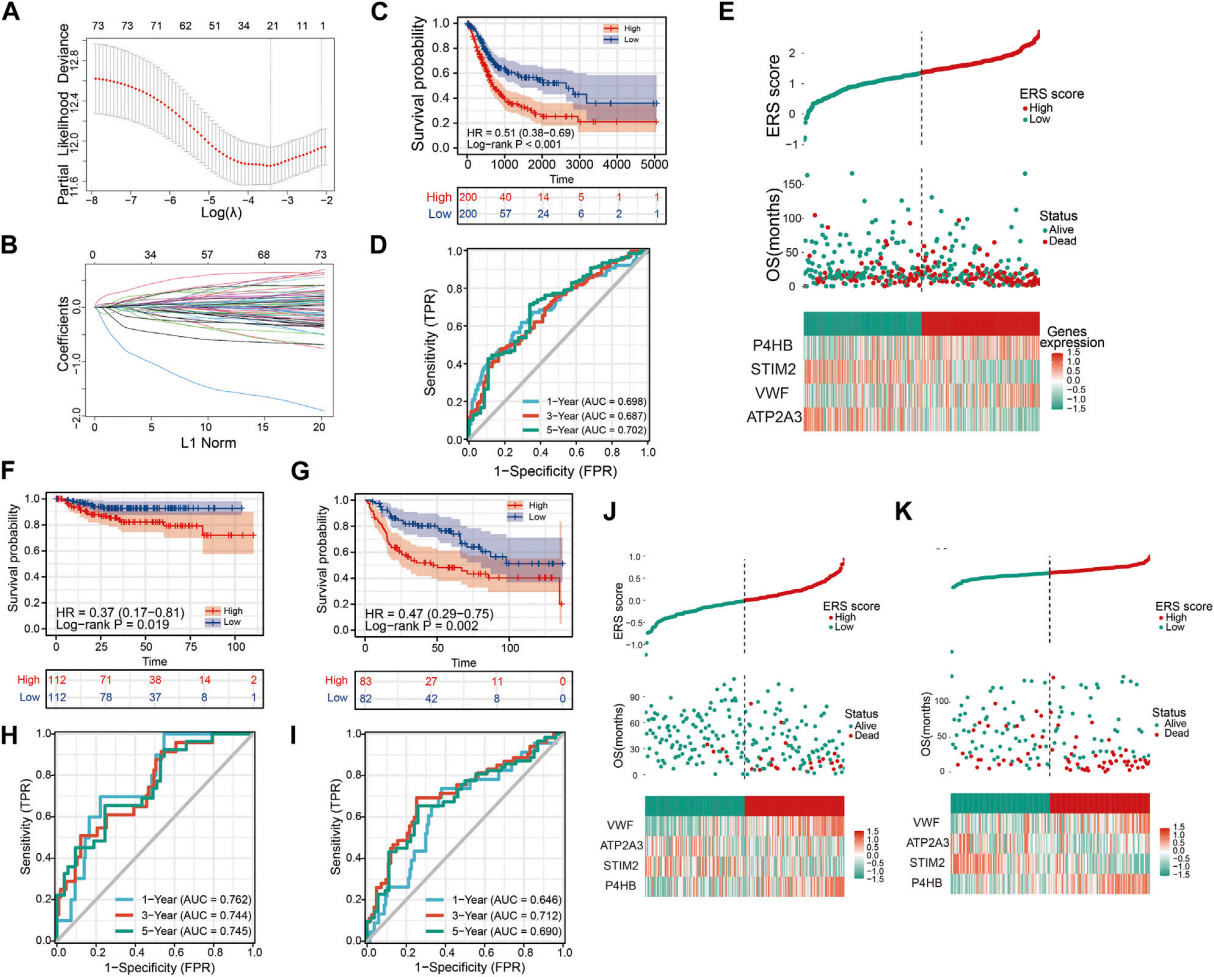
Constructing and validating the prognostic model. **(A, B)** LASSO regression analysis to remove covariance. **(C)** OS Kaplan-Meier curve for training cohort TCGA. **(D)** OS ROC curve for training cohort TCGA. **(E)** High and low ERS scores and heat map showing the expression of four genes in the prognostic model for training cohort TCGA. **(F, G)** OS Kaplan-Meier curve for verification cohorts GSE32894 and GSE13507. **(H, I)** OS ROC curve for verification cohorts GSE32894 and GSE13507. **(J, K)** High and low ERS scores and heat map showing the expression of four genes in the prognostic model for verification cohorts GSE32894 and GSE13507.

To better assess the prognosis of BLCA patients for clinical benefit, we constructed a ERS related prognostic signature in TCGA training database. The 258 ERS-related genes were analyzed by Univariate Cox analysis and 75 prognosis-related genes were obtained (Supplementary Figure S5). After LASSO analysis, 21 genes were included in the Multivariable Cox analysis (Figures 6A, B). Ultimately, we developed the 4-gene prognostic signature. ERS score=−ATP2A3*0.29859−STIM2*0.58523+VWF*0.31260+P4HB*0.35490. We divided the sample into high ERS score and low ERS score groups according to the median scores (Figure 6E). Prognosis was predicted by Kaplan-Meier curves for OS in high and low ERS score groups (Figure 6C). The results showed that patients in the high ERS score group had a worse prognosis (*p* < 0.001). For the OS survival prediction of ERS signature, the AUC of the ROC curves for 1, 3 and 5, years were 0.698, 0.687, and 0.702 (Figure 6D). It demonstrated that ERS signature we constructed has strong predictive value for OS prognosis in the training set.

### Validation of prognostic signature

To further demonstrate that ERS scores could steadily predict prognosis, two validation sets GSE32894 and GSE13507 were integrated in our study. Similarly, we also divided the sample into high and low ERS score groups according to the median of the ERS scores (Figures 6J, K). The OS Kaplan-Meier curves for both validation sets indicate that high ERS score group has the worse OS prognosis (Figures 6F, G). The AUC of the ROC curves for 1, 3, and 5 years in the GSE32894 were 0.762, 0.744, and 0.745 (Figure 6H). The AUC of the ROC curves for 1, 3, and 5 years in the GSE13507 were 0.646, 0.712, and 0.690 (Figure 6I). The results were evidence of the powerful prognostic capability of the ERS signature.

### Construction of Nomogram

**FIGURE 7:**
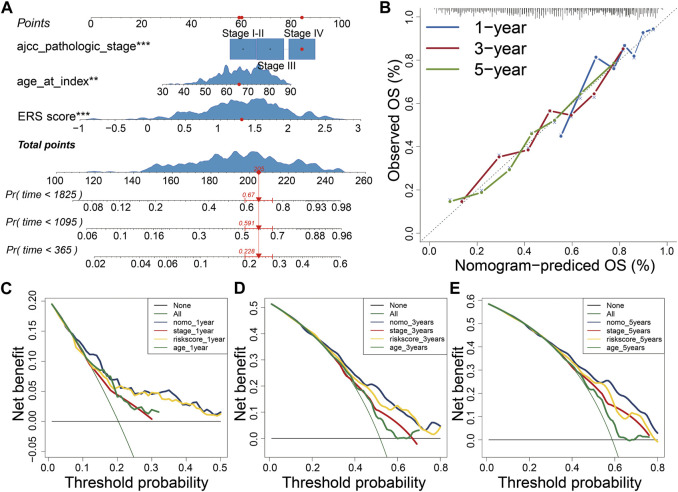
Construction of Nomogram. **(A)** Constructing the Nomogram to assess patient prognosis. **(B–E)** Calibration plots and decision curves for 1-, 3-, and 5-year survival projections.

Further, we explored the correlation between ERS score and clinical characteristics. Univariate and Multivariable Cox analysis showed that ERS score, stage and age were independent risk factors for the prognosis of BLCA (Supplementary Figures S6A, B). In addition, ERS scores were higher in patients older than 70 years, with higher stage, poorer OS prognosis, higher T-stage, and higher N-stage. However, there were no statistically significant differences in ERS scores by gender and M-stage (Supplementary Figures S6C–I). A clinically stratified analysis of gender, age, stage, T-stage, N-stage, and M-stage was performed in the TCGA database to analyze ERS signature prognostic performance. The results showed that patients in the low ERS scores cohort had improved survival outcomes compared with those in the high ERS score cohort in gender, age, stage, T-stage, and N0 (*p* < 0.05, Supplementary Figures S7A–I). In contrast, no prognostic differences were observed between the low and high ERS score cohort in the clinical stratification of N1-3 and M stage (Supplementary Figures S7J–L). To enhance clinical applicability, we constructed nomogram using stage, age, and scores (Figure 7A). Calibration plots and decision curves for 1-, 3-, and 5-year survival prediction indicated that nomogram has good predictive accuracy and benefit (Figures 7B–E).

### TME and immunotherapy prediction of two groups

**FIGURE 8:**
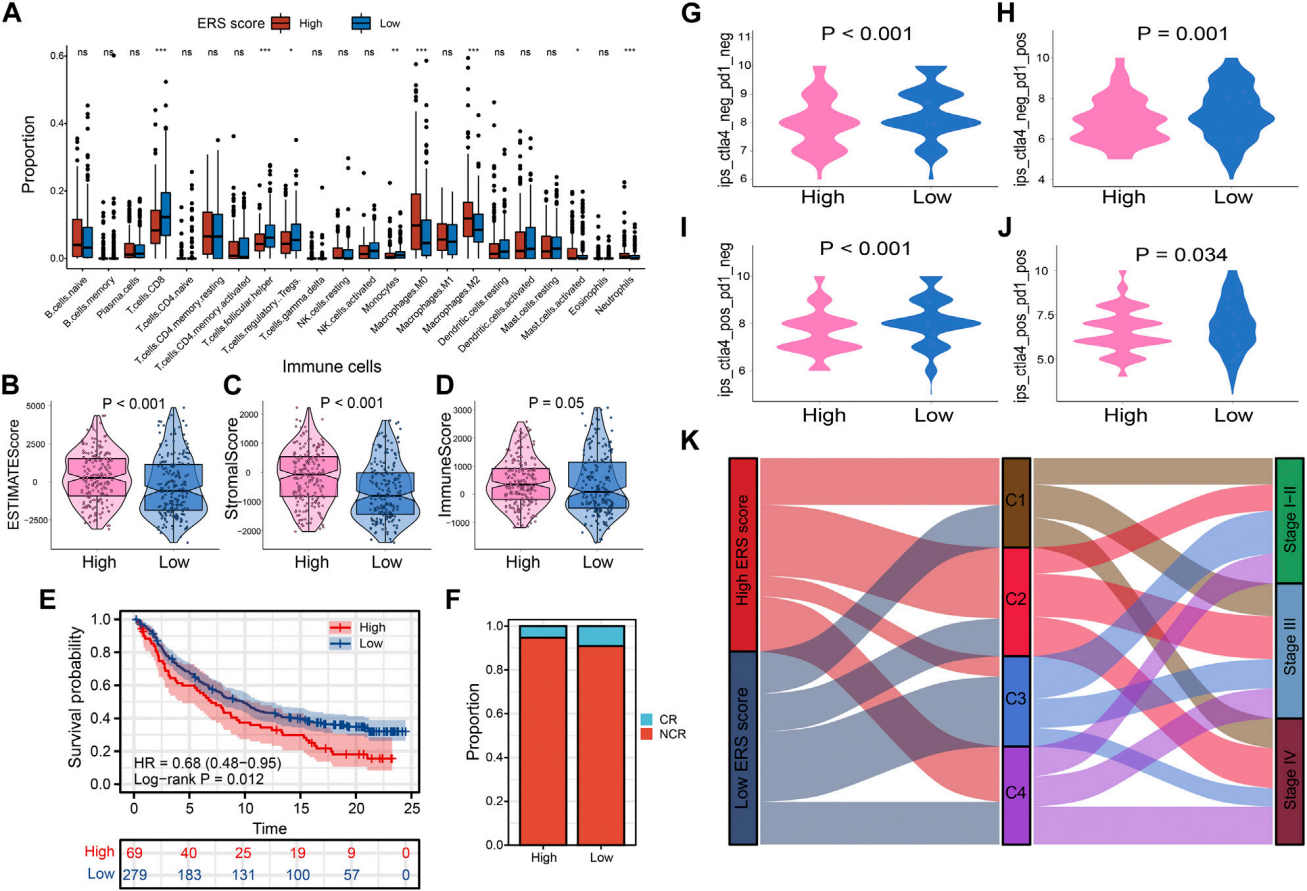
Prognostic signatures of tumor immune microenvironment and drug prediction of two groups. **(A)** Immune cell infiltration analysis assessed by Cibersort algorithm of two groups. **(B–D)** Comparison of estimate scores, immune scores and stromal scores in two groups. **(E, F)** The IMvigor210 cohort assesses effectiveness of PD-L1 blockage therapy. **(G–J)** Violin chart showing IPS score differences. **(K)** The correlation between the ERS scores, the four subtypes, and the pathological stage is shown in the Sankey diagram.

To explore the characteristics between the high and low ERS score groups, we explored the differences in immune microenvironment and immunotherapy effects between the two groups. M0 macrophages, M2 macrophages, mast cells, and neutrophil infiltration were evident in the high ERS score group. In contrast, immune cells with tumor-killing properties such as CD8T cells was enriched in low-ERS score group (Figure 8A). The estimate score, immune score and stromal score were also higher in high ERS score group than in low ERS score group (Figures 8B–D). To evaluate the effect of immunotherapy, we introduced the IMvigor210 cohort and the IPS score for the study. The results showed that the prognosis and complete response rate of patients in low ERS score group were better than those in high ERS score group after PD-L1 blockage therapy (Figures 8E, F). Furthermore, IPS scores, IPS-CTLA4 blocker scores, IPS-PD1 blocker scores, and IPS-CTLA4 and PD1 blocker scores were higher in samples with low ERS scores (*p* < 0.05; Figures 8G–J), indicating that BLCA samples with low ERS scores may be suitable for anti PD-1 and CTLA-4 immunotherapy. Inspiringly, we were surprised to find that the ability of the ERS signature to predict immunotherapy efficacy was robust in our TMU-BLCA cohort. Patients with low ERS scores had good immunotherapy outcomes. These results further confirm the clinical importance of the ERS signature (Supplementary Figure S10F). Finally, we found that most patients in the high ERS score cohort were associated with C2 and advanced stage, whereas patients with low ERS score were C3 and earlier stage (Figure 8K).

### Single cell analysis

Through TISCH website, we accessed to three single-cell databases GSE130001, GSE145281 and GSE149652 (Supplementary Figures S8A–C). P4HB expressed in a variety of cells, mainly expressed in stromal cells, including endothelial, fibroblasts, myofibroblasts and epithelial (Supplementary Figures S8D, S9A). VWF mainly expressed in endothelial (Supplementary Figures S8E, S9B). ATP2A3 mainly expressed in immune cells such as B-cell, while STIM2 expressed in a few amounts in a variety of cells (Supplementary Figures S8F–G, S9C–D).

### The ERS signature genes expression validation by qRT-PCR

We verified the expression patterns of ERS model genes (ATP2A3, STIM2, VWF, and P4HB) in BLCA cell lines and normal uroepithelial cell lines by RT-qPCR (Supplementary Figure S10A). Compared with the expression levels in SV-HUC-1 uroepithelial cells, ATP2A3 and VWF were meaningfully low expressed in 4 bladder cancer cell lines (T24, 253J-BV, EJ, UMUC3), while P4HB were high. STIM2 were low expressed in T24, 253J-BV and UMUC3 cell lines but not significant in EJ cells. In addition, we found downregulated expression of ATP2A3, VWF in tumor tissues (Supplementary Figures S10B–E). Conversely, P4HB were upregulated. The above results implied that ERS model genes may play an important potential role in bladder cancer progression.

## Discussion

Current research on cancer has shifted the attention from individual tumor cells to the environment in which tumor cells arise and live. The environment, as known for us called TME, includes not only the core tumor cells, but also various immune cells, fibroblasts, extracellular matrix, and multiple signaling molecules that infiltrate it. There have been numerable studies that have confirmed that various components of the tumor microenvironment can inhibit the killing of tumor cells by immune cells, promote the proliferation and metastasis of tumor cells, and also can develop resistance to chemotherapeutic drugs, immunotherapeutic drugs (Kaymak et al., 2021). Therefore, a total commitment to the study of the immune microenvironment is of great significances for the treatment of cancer (Wu and Dai, 2017).

Up to date, a large number of studies have been reported that endoplasmic reticulum stress is closely associated with cancer development (Cubillos-Ruiz et al., 2017). Under normal conditions, the endoplasmic reticulum can process and fold proteins. However, in the tumor microenvironment, the presence of multiple stressors such as hypoxia, low PH and disorders of nutritional supply can lead to the appearance of a large number of unfolded or misfolded proteins in both malignant and stromal cells, which results in a state of ERS. Cells that are unable to tolerate this state undergo apoptosis, autophagy or immunogenic death. But what is inconceivable is that if tolerated by unfolded protein response (UPR) pathway, it may promote malignant development through a variety of mechanisms including cellular reprogramming (Chen and Cubillos-Ruiz, 2021). In addition, endoplasmic reticulum stress leads to chemoresistance as well as suppression of antitumor immunity (Song and Cubillos-Ruiz, 2019). In bladder cancer, H. H. Zhang et al. Zhang reported that OTUB1 can maintain ATF6 expression by inhibits the ubiquitination process thus promote progression (Tadros et al., 2017; Zhang et al., 2021). There are currently some studies targeting specific endoplasmic reticulum stress-related genes in bladder cancer (Nie et al., 2021), but comprehensive analyses of ERS-mediated TME in BLCA are rare. Identifying different ERS response patterns will help us understand the underlying mechanisms of endoplasmic reticulum stress and the tumor microenvironment, and will also allow us to derive appropriate drugs for precision therapy.

In our research, we first performed consensus clustering of 400 TCGA BLCA samples into 4 clusters by ERS-related genes. These four clusters exhibited different prognosis, degree of biological pathway enrichment, TME, and mutation status. ERS-related pathways were enriched in C1 and C2 with poorer survival, suggesting activation of ERS process was associated with short-term survival time. Similarly, hypoxia-related pathways were enriched in both clusters, suggesting that the occurrence of ERS in BLCA might mediated by hypoxia in TME. Besides these, C2 was characterized by poorest prognose, advanced tumor stage, basal subtype of BLCA, activation of stroma-associated and immune-related pathways, high mutation burden of TP53, as well as high immune cell infiltration level, particularly M2 macrophages. In accordance with previous researches, high immune infiltration level tends to a better prognose, which went against our results. Through further analysis we speculate that the poorer prognosis may be due to the overwhelmingly high expression of immune checkpoints in the C2 subtype, which implies immune escape and may result in the inability of immune cells to function effectively. Additionally, there have been studies shown that tumor-killing immune cells, such as T-cells, require proper targeting and migration to maximize their effects (Salmon et al., 2012). However, due to activation of the C2 subset of matrix-associated pathways, immune cells may not be able to reach the core of the tumor and thus fail to act as tumor killers. Moreover, M2 macrophages presented in the immune microenvironment abundantly of C2 can secrete immunosuppressive factors such as TGF-β and IL-10, which weaken the effects of T-cells, NK cells and other tumor-killing cells (Komohara et al., 2016). C1 showed lower level of immune infiltration second only to C2 and high tumor specific pathways activation such as MTOR, p53 and ERBB signaling pathways. Differently, C3 was featured as low immune and stromal scores, high mutation burden of FGFR3, luminal subtypes of BLCA, activation of fatty acid metabolism pathway, early stage of tumor, as well as a highest survival rate. Therefore, we hypothesized that tumorigenesis in C3 may be related to fatty acid metabolism in the TME. However, C4 was high correlation with DNA damage repair and high level of TMB, this may be the reason why C4 has a better prognosis. We validated the above crucial results with the GSE32894 dataset, and the results further confirmed the stability and reliability of the ERS clusters for the BLCA phenotype in our study. In order to achieve precise treatment, we further evaluated the cluster for which chemotherapy and immunotherapy are suitable. Common chemotherapy drugs for BLCA such as Cisplatin, Gemcitabine, Paclitaxel, Doxorubicin were suitable for C1 and C2, instead C3 or C4. TMB and TIDE score were used to predict immunotherapy sensitivity, and the results showed that patients in C4 may be more suitable for immunotherapy. TMB has been shown to be a good predictor of immunotherapy in an existing study (Gibney et al., 2016). The above results provide great help to our understanding of TME with ERS in BLCA, and are very instructive for clinical purposes.

According to the ERS clusters we developed, patients in different ERS cluster have different survival time, so we consider that ERS status may have a profound impact on prognosis. It has been demonstrated that ERS is associated with prognosis of patients with bladder cancer. For example, overexpression of XBP1 is associated with poorer OS in patients with metastatic cell carcinoma (Chen et al., 2016). However, there are no studies specifically predicting the prediction of patient prognosis and clinical characteristics in BLCA. Therefore, it is necessary to develop ERS-related prognostic signature to guide clinicians to implement individualized treatment. In our study, we developed a 4-gene prognostic model for ERS-related genes by lasso-cox analysis in TCGA cohort and validated in GSE13507 and GSE32894. Higher ERS score meant poorer prognosis. We then correlated the ERS scores with clinical characteristics, and the results further confirmed the far-reaching clinical implications of our signature. Surprisingly, we found that patients with high ERS scores were significantly associated with the C2 cluster, and conversely, patients with low ERS scores were C3 cluster. In addition, the results of our analysis of Imvigor210, TMU-BLCA cohorts and IPS score showed us patients of low ERS score group may more suitable for immune checkpoint inhibitors, which further verified the above results. Finally, we briefly compared several previous bladder cancer models and found that the ROC curves of our ERS signature were significantly better than theirs, which illustrated that the prognostic of ERS to predict patients with bladder cancer is robust (Liang et al., 2021; Yang et al., 2021; Wang et al., 2022). In addition, we validated the ERS model to predict immunotherapy response using a real-world cohort and obtained the ideal results. The results further validate that ERS model has important clinical implications.

Our study provided a comprehensive and systematic analysis of the transcriptome profile associated with endoplasmic reticulum stress, and on the basis of this, a prognostic model was developed to guide clinical personalized treatment. However, there are still some limitations in our study. For example, we need to further expand the sample size to prove the reliability of ERS clusters. Additionally, our study was retrospective and prospective studies are needed to further confirm the reliability of the findings.

## Conclusion

In conclusion, the novel ERS clusters we established reflect to some extent the underlying mechanisms of TME in BLCA and provides new insights for personalized treatment of bladder cancer. Meanwhile, the ERS signature we developed is equally significant for guiding the prognosis of BLCA patients.

## Data Availability

The original contributions presented in the study are included in the article/Supplementary Material, further inquiries can be directed to the corresponding author.

## Supplementary material

SUPPLEMENTARY FIGURE S1 Association of ERS subtypes with different clinical characteristics (A–D: Age, N stage, Pathological stage and T stage).

SUPPLEMENTARY FIGURE S2 Critical characteristics of different ERS subtypes in validation set. (A) Four subtypes of cancer immunity cycle. (B) Differential enrichment of M2 macrophages in four subtypes. (C) Molecular subtypes of BLCA in different ERS subgroups. (D) Association of Pathological T stage with ERS subtypes. (E) The heat map shows the metabolism-related pathways for the 4 subtypes.

SUPPLEMENTARY FIGURE S3 Immune infiltration and immunomodulators analysis in validation set. (A–B) Heat map showing the results of 7 methods to assess immune infiltration and differential expression of immunomodulator genes in the four subtypes.

SUPPLEMENTARY FIGURE S4 Drug sensitivity analysis. (A–D) Comparison of IC50 of four chemotherapy drugs in four subtypes.

SUPPLEMENTARY FIGURE S5 Univariant cox regression analysis of ERS-related genes.

SUPPLEMENTARY FIGURE S6 The significance of ERS scores in clinical characteristics. (A–B) Univariant and multivariant cox analysis of clinical characteristics and ERS score. (C–I) ERS scores in different clinical subgroups (Age, gender, pathological stage, vital status, T stage, N stage, M stage) illustrated by box plots.

SUPPLEMENTARY FIGURE S7 Survival analysis for multiple clinical subgroups is presented with K–M curves. (A–B) Gender; (C–D) Age; (E–F) pathological stage; (G–H) T stage; (I–J) N stage; (K–L) M stage.

SUPPLEMENTARY FIGURE S8 Exploration of model genes expressed in what kind of cells by single-cell analysis. (A–C) UMAPs plots of GSE130001, GSE145281, GSE149652. (D–G) UMAP plots of 4 signature genes in above-mentioned databases.

SUPPLEMENTARY FIGURE S9 Quantification of 4 signature genes expression levels in different cells. (A–D) P4HB, VWF, ATP2A3, STIM2.

SUPPLEMENTARY FIGURE S10 (A) Detection of ERS signature gene expression levels in using RT-qPCR in 4 BLCA cell lines (T24, 253J-BV, EJ, UMUC3) and normal uroepithelial cell line (SV-HUC-1). (B–E) Further validation of ERS gene expression levels in paired tumors and adjacent normal tissues. (F) Validation the ability of ERS signature to predict immunotherapy efficacy in the TMU-BLCA cohort.

## Abbreviations

AUC, Area under the cure; BLCA, bladder cancer; EMT, Epithelial mesenchymal transition; ERS, endoplasmic reticulum stress; GEO, Gene Expression Omnibus; IPS, Immunophenoscore; ROC, Receiver operating characteristic curve; TCGA, The Cancer Genome Atlas; TIDE, Tumor Immune Dysfunction and Exclusion; TMB, Tumor mutation burden; TME, Tumor microenvironment; UPR, Unfolded protein response.

## References

[B1] Abd El-Salam M. A. Smith C. E. P. Pan C. X. (2022). Insights on recent innovations in bladder cancer immunotherapy. Cancer Cytopathol. 130, 667–683. 10.1002/cncy.22603 35653623

[B2] Afonso J. Santos L. L. Longatto-Filho A. Baltazar F. (2020). Competitive glucose metabolism as a target to boost bladder cancer immunotherapy. Nat. Rev. Urol. 17, 77–106. 10.1038/s41585-019-0263-6 31953517

[B3] Aran D. Hu Z. Butte A. J. (2017). xCell: digitally portraying the tissue cellular heterogeneity landscape. Genome Biol. 18, 220. 10.1186/s13059-017-1349-1 29141660 PMC5688663

[B4] Barrett T. Wilhite S. E. Ledoux P. Evangelista C. Kim I. F. Tomashevsky M. (2013). NCBI GEO: Archive for functional genomics data sets--update. Nucleic Acids Res. 41, D991–D995. 10.1093/nar/gks1193 23193258 PMC3531084

[B5] Becht E. Giraldo N. A. Lacroix L. Buttard B. Elarouci N. Petitprez F. (2016). Estimating the population abundance of tissue-infiltrating immune and stromal cell populations using gene expression. Genome Biol. 17, 218. 10.1186/s13059-016-1070-5 27765066 PMC5073889

[B6] Blum A. Wang P. Zenklusen J. C. (2018). SnapShot: TCGA-analyzed tumors. Cell 173, 530. 10.1016/j.cell.2018.03.059 29625059

[B7] Charoentong P. Finotello F. Angelova M. Mayer C. Efremova M. Rieder D. (2017). Pan-cancer immunogenomic analyses reveal genotype-immunophenotype relationships and predictors of response to checkpoint blockade. Cell Rep. 18, 248–262. 10.1016/j.celrep.2016.12.019 28052254

[B8] Chen X. Cubillos-Ruiz J. R. (2021). Endoplasmic reticulum stress signals in the tumour and its microenvironment. Nat. Rev. Cancer 21, 71–88. 10.1038/s41568-020-00312-2 33214692 PMC7927882

[B9] Chen D. S. Mellman I. (2013). Oncology meets immunology: The cancer-immunity cycle. Immunity 39, 1–10. 10.1016/j.immuni.2013.07.012 23890059

[B10] Chen W. Zhou J. Wu K. Huang J. Ding Y. Yun E. J. (2016). Targeting XBP1-mediated β-catenin expression associated with bladder cancer with newly synthetic Oridonin analogues. Oncotarget 7, 56842–56854. 10.18632/oncotarget.10863 27472396 PMC5302956

[B11] Choi W. Porten S. Kim S. Willis D. Plimack E. R. Hoffman-Censits J. (2014). Identification of distinct basal and luminal subtypes of muscle-invasive bladder cancer with different sensitivities to frontline chemotherapy. Cancer Cell 25, 152–165. 10.1016/j.ccr.2014.01.009 24525232 PMC4011497

[B12] Cubillos-Ruiz J. R. Bettigole S. E. Glimcher L. H. (2017). Tumorigenic and immunosuppressive effects of endoplasmic reticulum stress in cancer. Cell 168, 692–706. 10.1016/j.cell.2016.12.004 28187289 PMC5333759

[B13] Damrauer J. S. Hoadley K. A. Chism D. D. Fan C. Tiganelli C. J. Wobker S. E. (2014). Intrinsic subtypes of high-grade bladder cancer reflect the hallmarks of breast cancer biology. Proc. Natl. Acad. Sci. U. S. A. 111, 3110–3115. 10.1073/pnas.1318376111 24520177 PMC3939870

[B14] Finotello F. Mayer C. Plattner C. Laschober G. Rieder D. Hackl H. (2019). Molecular and pharmacological modulators of the tumor immune contexture revealed by deconvolution of RNA-seq data. Genome Med. 11, 34. 10.1186/s13073-019-0638-6 31126321 PMC6534875

[B15] Gibney G. T. Weiner L. M. Atkins M. B. (2016). Predictive biomarkers for checkpoint inhibitor-based immunotherapy. Lancet Oncol. 17, e542–e551. 10.1016/S1470-2045(16)30406-5 27924752 PMC5702534

[B16] HäNZELMANN S. Castelo R. Guinney J. (2013). Gsva: Gene set variation analysis for microarray and RNA-seq data. BMC Bioinforma. 14, 7. 10.1186/1471-2105-14-7 PMC3618321 23323831

[B17] Jiang P. Gu S. Pan D. Fu J. Sahu A. Hu X. (2018). Signatures of T cell dysfunction and exclusion predict cancer immunotherapy response. Nat. Med. 24, 1550–1558. 10.1038/s41591-018-0136-1 30127393 PMC6487502

[B18] Kamoun A. de ReynièS A. Allory Y. SjöDAHL G. Robertson A. G. Seiler R. (2020). A consensus molecular classification of muscle-invasive bladder cancer. Eur. Urol. 77, 420–433. 10.1016/j.eururo.2019.09.006 31563503 PMC7690647

[B19] Kaymak I. Williams K. S. Cantor J. R. Jones R. G. (2021). Immunometabolic interplay in the tumor microenvironment. Cancer Cell 39, 28–37. 10.1016/j.ccell.2020.09.004 33125860 PMC7837268

[B20] Kim W. J. Kim E. J. Kim S. K. Kim Y. J. Ha Y. S. Jeong P. (2010). Predictive value of progression-related gene classifier in primary non-muscle invasive bladder cancer. Mol. Cancer 9, 3. 10.1186/1476-4598-9-3 20059769 PMC2821358

[B21] Komohara Y. Fujiwara Y. Ohnishi K. Takeya M. (2016). Tumor-associated macrophages: Potential therapeutic targets for anti-cancer therapy. Adv. Drug Deliv. Rev. 99, 180–185. 10.1016/j.addr.2015.11.009 26621196

[B22] Le T. Aronow R. A. Kirshtein A. Shahriyari L. (2021). A review of digital cytometry methods: Estimating the relative abundance of cell types in a bulk of cells. Brief. Bioinform 22, bbaa219. 10.1093/bib/bbaa219 33003193 PMC8293826

[B23] Li T. Fan J. Wang B. Traugh N. Chen Q. Liu J. S. (2017). TIMER: A web server for comprehensive analysis of tumor-infiltrating immune cells. Cancer Res. 77, e108–e110. 10.1158/0008-5472.CAN-17-0307 29092952 PMC6042652

[B24] Li Z. Wang Z. Yang S. Shen C. Zhang Y. Jiang R. (2022). CircSTK39 suppresses the proliferation and invasion of bladder cancer by regulating the miR-135a-5p/NR3C2-mediated epithelial-mesenchymal transition signaling pathway. Cell Biol. Toxicol. 10.1007/s10565-022-09785-3 36538242

[B25] Liang Y. Ye F. Xu C. Zou L. Hu Y. Hu J. (2021). A novel survival model based on a Ferroptosis-related gene signature for predicting overall survival in bladder cancer. BMC Cancer 21, 943. 10.1186/s12885-021-08687-7 34418989 PMC8380338

[B26] Liberzon A. Birger C. ThorvaldsdóTTIR H. Ghandi M. Mesirov J. P. Tamayo P. (2015). The Molecular Signatures Database (MSigDB) hallmark gene set collection. Cell Syst. 1, 417–425. 10.1016/j.cels.2015.12.004 26771021 PMC4707969

[B27] Liu X. Viswanadhapalli S. Kumar S. Lee T. K. Moore A. Ma S. (2022). Targeting LIPA independent of its lipase activity is a therapeutic strategy in solid tumors via induction of endoplasmic reticulum stress. Nat. Cancer 3, 866–884. 10.1038/s43018-022-00389-8 35654861 PMC9325671

[B28] Mariathasan S. Turley S. J. Nickles D. Castiglioni A. Yuen K. Wang Y. (2018). TGFβ attenuates tumour response to PD-L1 blockade by contributing to exclusion of T cells. Nature 554, 544–548. 10.1038/nature25501 29443960 PMC6028240

[B29] Matsuo K. Gray M. J. Yang D. Y. Srivastava S. A. Tripathi P. B. Sonoda L. A. (2013). The endoplasmic reticulum stress marker, glucose-regulated protein-78 (GRP78) in visceral adipocytes predicts endometrial cancer progression and patient survival. Gynecol. Oncol. 128, 552–559. 10.1016/j.ygyno.2012.11.024 23200913 PMC4199224

[B30] Mayakonda A. Lin D. C. Assenov Y. Plass C. Koeffler H. P. (2018). Maftools: Efficient and comprehensive analysis of somatic variants in cancer. Genome Res. 28, 1747–1756. 10.1101/gr.239244.118 30341162 PMC6211645

[B31] Mo Q. Nikolos F. Chen F. Tramel Z. Lee Y. C. Hayashi K. (2018). Prognostic power of a tumor differentiation gene signature for bladder urothelial carcinomas. J. Natl. Cancer Inst. 110, 448–459. 10.1093/jnci/djx243 29342309 PMC6279371

[B32] Necchi A. Joseph R. W. Loriot Y. Hoffman-Censits J. Perez-Gracia J. L. Petrylak D. P. (2017). Atezolizumab in platinum-treated locally advanced or metastatic urothelial carcinoma: Post-progression outcomes from the phase II IMvigor210 study. Ann. Oncol. 28, 3044–3050. 10.1093/annonc/mdx518 28950298 PMC5834063

[B33] Nie Z. Chen M. Wen X. Gao Y. Huang D. Cao H. (2021). Endoplasmic reticulum stress and tumor microenvironment in bladder cancer: The missing link. Front. Cell Dev. Biol. 9, 683940. 10.3389/fcell.2021.683940 34136492 PMC8201605

[B34] Racle J. Gfeller D. (2020). Epic: A tool to estimate the proportions of different cell types from bulk gene expression data. Methods Mol. Biol. 2120, 233–248. 10.1007/978-1-0716-0327-7_17 32124324

[B35] Rebouissou S. Bernard-Pierrot I. de ReynièS A. Lepage M. L. Krucker C. Chapeaublanc E. (2014). EGFR as a potential therapeutic target for a subset of muscle-invasive bladder cancers presenting a basal-like phenotype. Sci. Transl. Med. 6, 244ra91. 10.1126/scitranslmed.3008970 25009231

[B36] Robertson A. G. Kim J. Al-Ahmadie H. Bellmunt J. Guo G. Cherniack A. D. (2017). Comprehensive molecular characterization of muscle-invasive bladder cancer. Cell 171, 540–556.e25. 10.1016/j.cell.2017.09.007 28988769 PMC5687509

[B37] Salmon H. Franciszkiewicz K. Damotte D. Dieu-Nosjean M. C. Validire P. Trautmann A. (2012). Matrix architecture defines the preferential localization and migration of T cells into the stroma of human lung tumors. J. Clin. Invest. 122, 899–910. 10.1172/JCI45817 22293174 PMC3287213

[B38] Salvagno C. Mandula J. K. Rodriguez P. C. Cubillos-Ruiz J. R. (2022). Decoding endoplasmic reticulum stress signals in cancer cells and antitumor immunity. Trends Cancer 8, 930–943. 10.1016/j.trecan.2022.06.006 35817701 PMC9588488

[B39] Siegel R. L. Miller K. D. Fuchs H. E. Jemal A. (2022). Cancer statistics, 2022. CA Cancer J. Clin. 72, 7–33. 10.3322/caac.21708 35020204

[B40] SjöDAHL G. Lauss M. LöVGREN K. Chebil G. Gudjonsson S. Veerla S. (2012). A molecular taxonomy for urothelial carcinoma. Clin. Cancer Res. 18, 3377–3386. 10.1158/1078-0432.CCR-12-0077-T 22553347

[B41] SjöDAHL G. Eriksson P. Patschan O. Marzouka N. A. Jakobsson L. Bernardo C. (2020). Molecular changes during progression from nonmuscle invasive to advanced urothelial carcinoma. Int. J. Cancer 146, 2636–2647. 10.1002/ijc.32737 31609466 PMC7079000

[B42] So J. S. (2018). Roles of endoplasmic reticulum stress in immune responses. Mol. Cells 41, 705–716. 10.14348/molcells.2018.0241 30078231 PMC6125421

[B43] Song M. Cubillos-Ruiz J. R. (2019). Endoplasmic reticulum stress responses in intratumoral immune cells: Implications for cancer immunotherapy. Trends Immunol. 40, 128–141. 10.1016/j.it.2018.12.001 30612925

[B44] Sun D. Wang J. Han Y. Dong X. Ge J. Zheng R. (2021). Tisch: A comprehensive web resource enabling interactive single-cell transcriptome visualization of tumor microenvironment. Nucleic Acids Res. 49, D1420–d1430. 10.1093/nar/gkaa1020 33179754 PMC7778907

[B45] Tadros S. Shukla S. K. King R. J. Gunda V. Vernucci E. Abrego J. (2017). De novo lipid synthesis facilitates gemcitabine resistance through endoplasmic reticulum stress in pancreatic cancer. Cancer Res. 77, 5503–5517. 10.1158/0008-5472.CAN-16-3062 28811332 PMC5645242

[B46] Thorsson V. Gibbs D. L. Brown S. D. Wolf D. Bortone D. S. Ou Yang T. H. (2018). The immune landscape of cancer. Immunity 48, 812–830.e14. 10.1016/j.immuni.2018.03.023 29628290 PMC5982584

[B47] Urra H. Dufey E. Avril T. Chevet E. Hetz C. (2016). Endoplasmic reticulum stress and the hallmarks of cancer. Trends Cancer 2, 252–262. 10.1016/j.trecan.2016.03.007 28741511

[B48] Van Rhijn B. W. G. Mertens L. S. Mayr R. Bostrom P. J. Real F. X. Zwarthoff E. C. (2020). FGFR3 mutation status and FGFR3 expression in a large bladder cancer cohort treated by radical cystectomy: Implications for anti-FGFR3 treatment?(†). Eur. Urol. 78, 682–687. 10.1016/j.eururo.2020.07.002 32682615

[B49] Wang Y. Zhang H. Hu X. (2022). Characterization of epithelial-mesenchymal transition identifies a gene signature for predicting clinical outcomes and therapeutic responses in bladder cancer. Dis. Markers 2022, 9593039. 10.1155/2022/9593039 36457546 PMC9708359

[B50] Wei C. Y. Zhu M. X. Zhang P. F. Huang X. Y. Wan J. K. Yao X. Z. (2022). PKCα/ZFP64/CSF1 axis resets the tumor microenvironment and fuels anti-PD1 resistance in hepatocellular carcinoma. J. Hepatol. 77, 163–176. 10.1016/j.jhep.2022.02.019 35219791

[B51] Wilkerson M. D. Hayes D. N. (2010). ConsensusClusterPlus: A class discovery tool with confidence assessments and item tracking. Bioinformatics 26, 1572–1573. 10.1093/bioinformatics/btq170 20427518 PMC2881355

[B52] Wu T. Dai Y. (2017). Tumor microenvironment and therapeutic response. Cancer Lett. 387, 61–68. 10.1016/j.canlet.2016.01.043 26845449

[B53] Yang W. Soares J. Greninger P. Edelman E. J. Lightfoot H. Forbes S. (2013). Genomics of drug sensitivity in cancer (GDSC): A resource for therapeutic biomarker discovery in cancer cells. Nucleic Acids Res. 41, D955–D961. 10.1093/nar/gks1111 23180760 PMC3531057

[B54] Yang L. Li C. Qin Y. Zhang G. Zhao B. Wang Z. (2021). A novel prognostic model based on ferroptosis-related gene signature for bladder cancer. Front. Oncol. 11, 686044. 10.3389/fonc.2021.686044 34422642 PMC8378228

[B55] Yu G. Wang L. G. Han Y. He Q. Y. (2012). clusterProfiler: an R package for comparing biological themes among gene clusters. Omics 16, 284–287. 10.1089/omi.2011.0118 22455463 PMC3339379

[B56] Zhang H. H. Li C. Ren J. W. Liu L. Du X. H. Gao J. (2021). OTUB1 facilitates bladder cancer progression by stabilizing ATF6 in response to endoplasmic reticulum stress. Cancer Sci. 112, 2199–2209. 10.1111/cas.14876 33686769 PMC8177800

