# Supplementary figures and images for: Comprehensive characterization of endoplasmic reticulum stress in bladder cancer revealing the association with tumor immune microenvironment and prognosis
Source: Front Genet. 2023 Apr 7;14:1097179. doi: 10.3389/fgene.2023.1097179 (PMC10119429; doi:10.3389/fgene.2023.1097179)

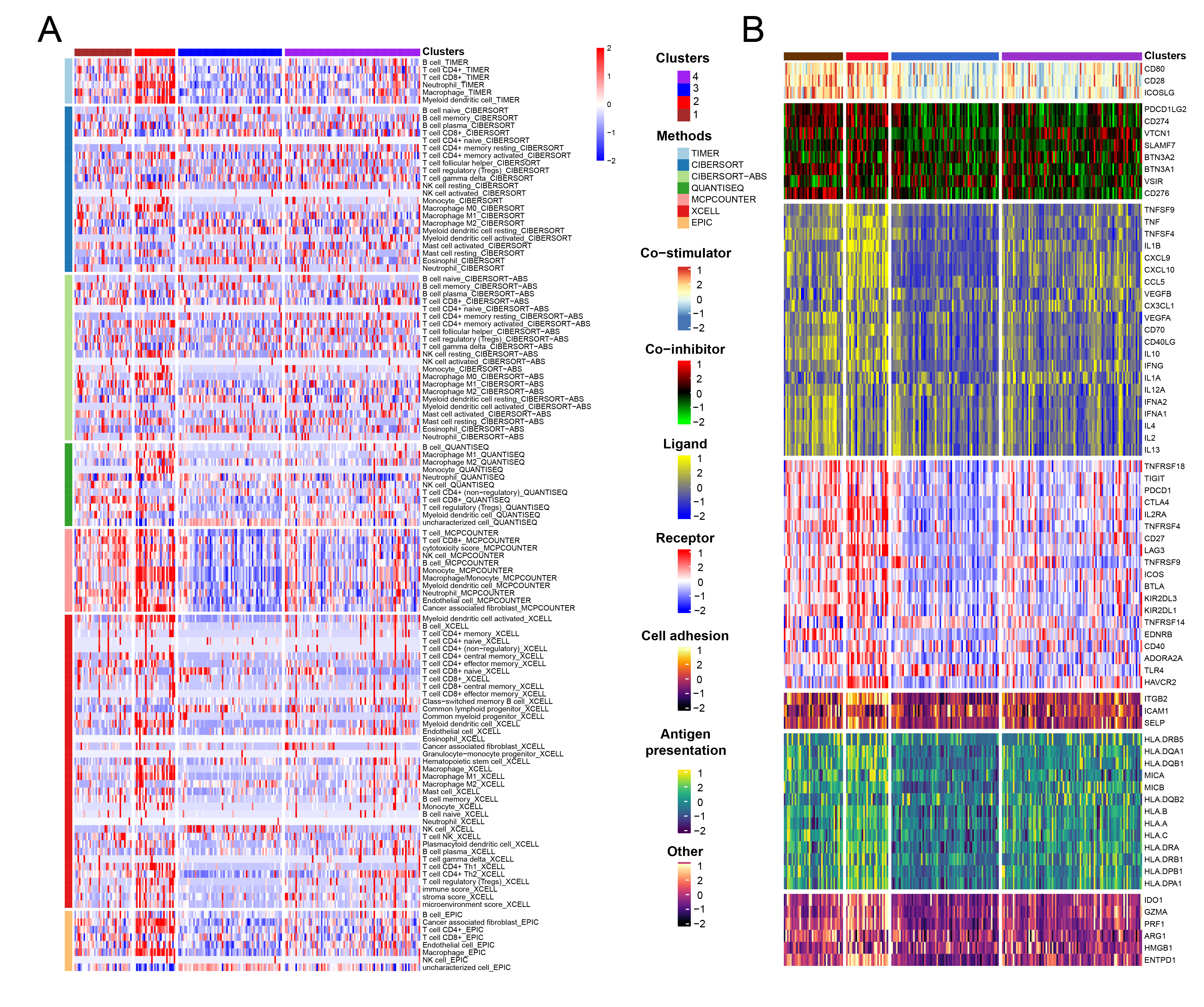

Supplement: Supplementary file 1 [file Image3.JPEG]

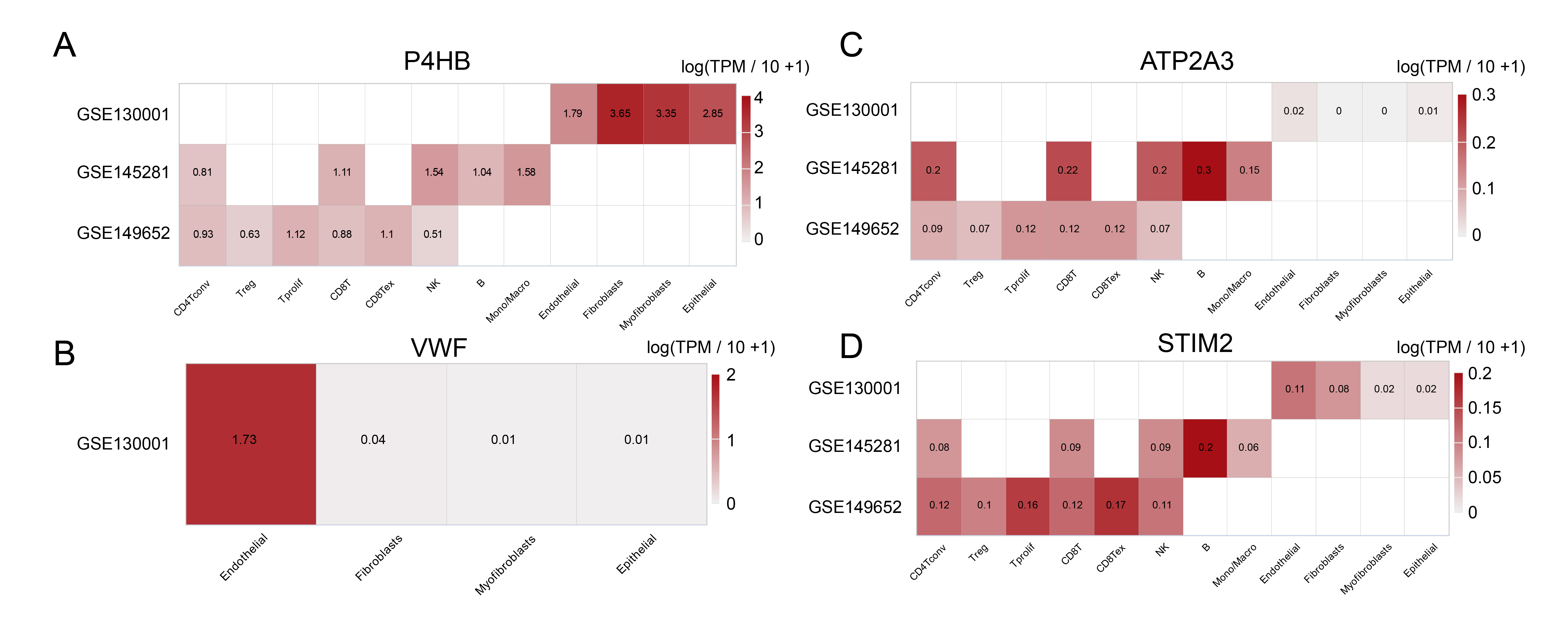

Supplement: Supplementary file 5 [file Image9.JPEG]

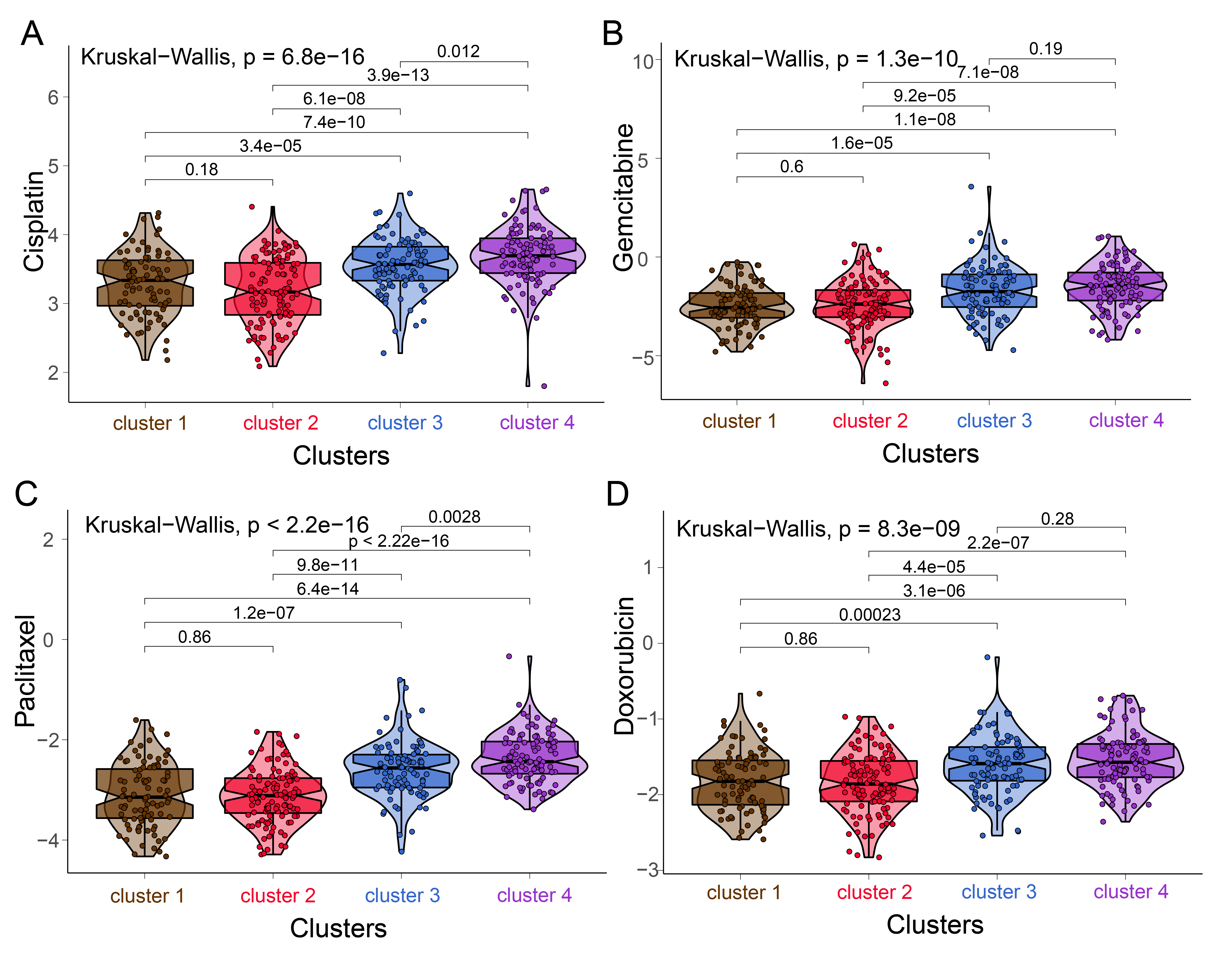

Supplement: Supplementary file 6 [file Image4.TIF]

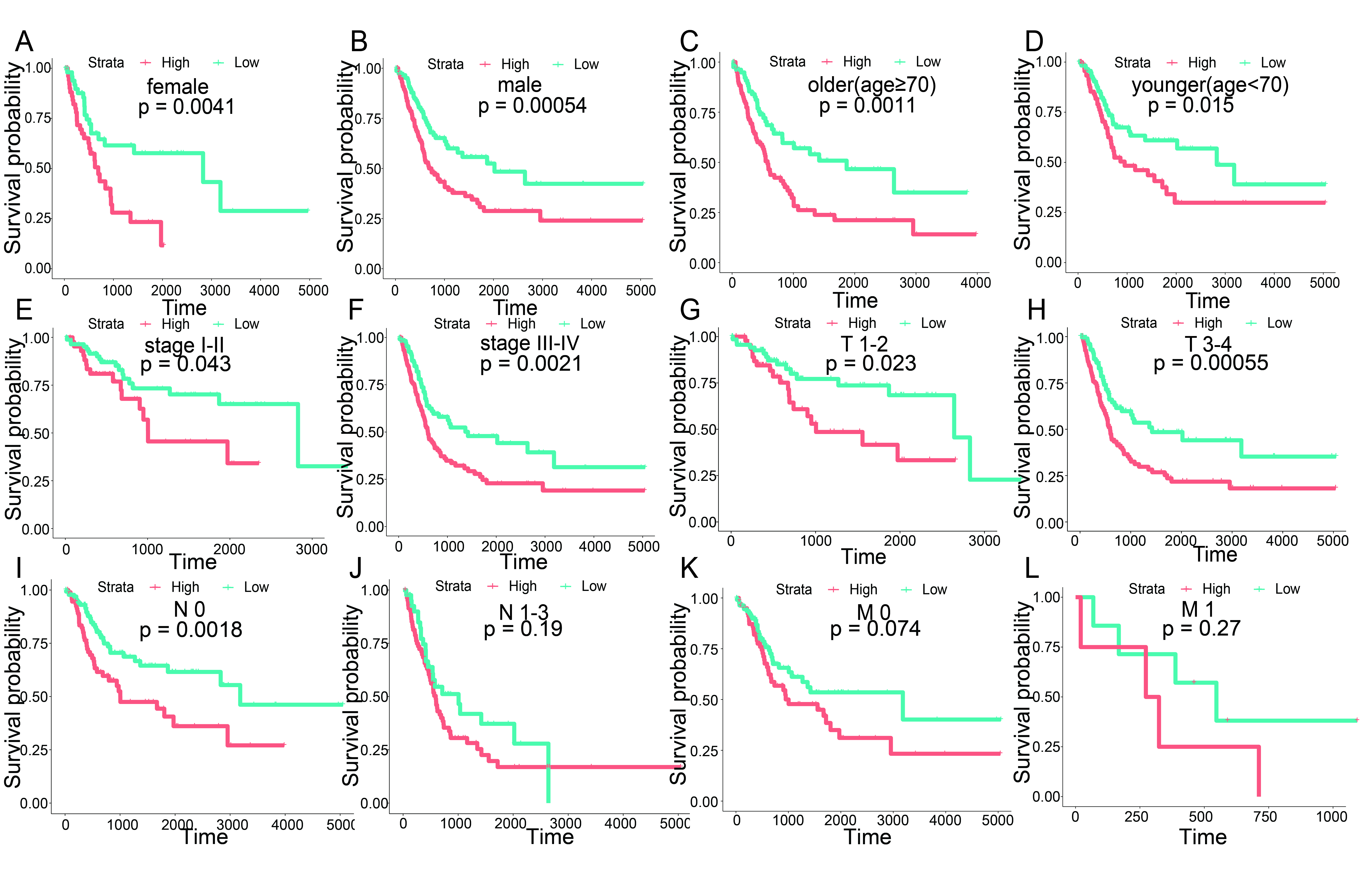

Supplement: Supplementary file 7 [file Image7.JPEG]

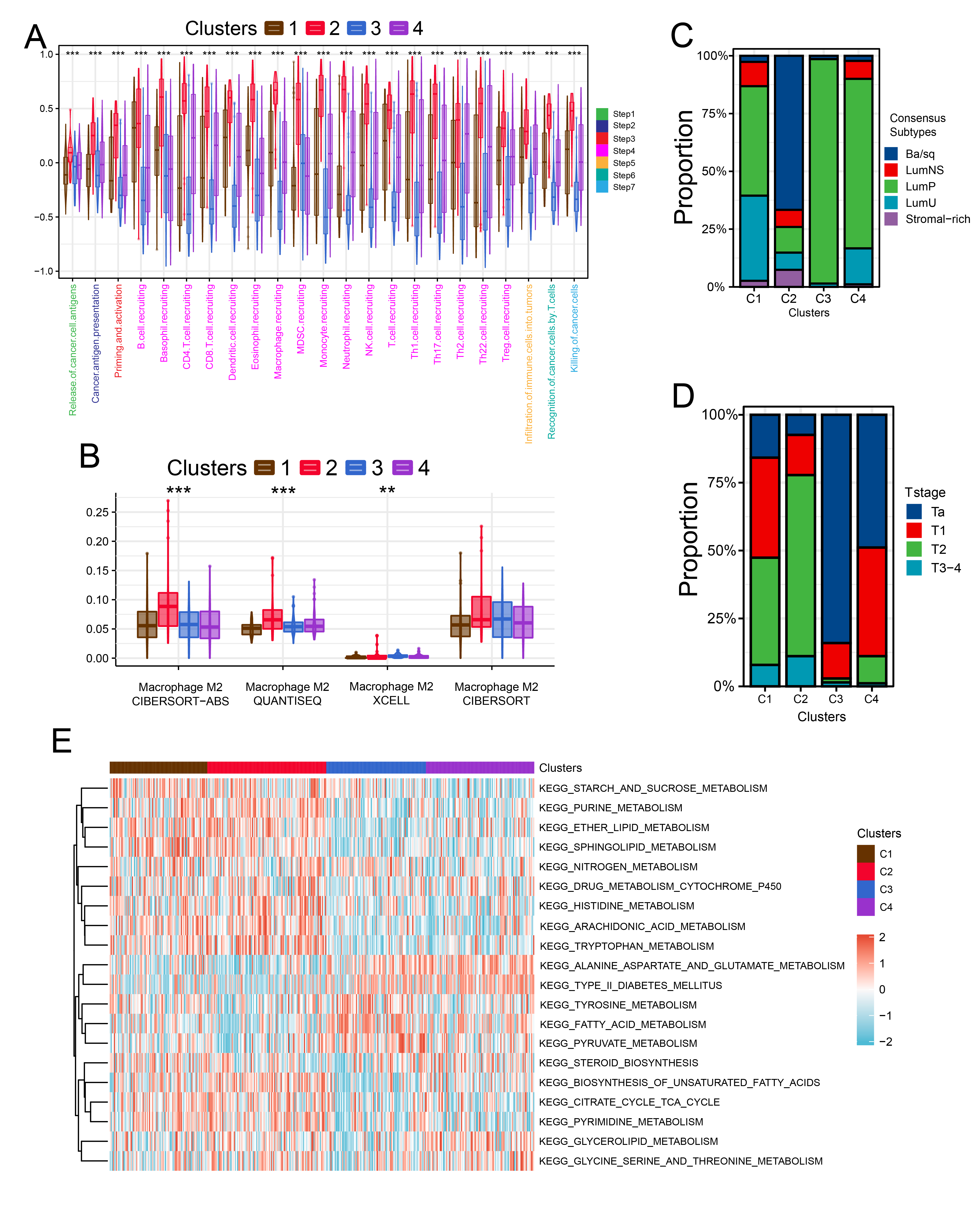

Supplement: Supplementary file 8 [file Image2.JPEG]

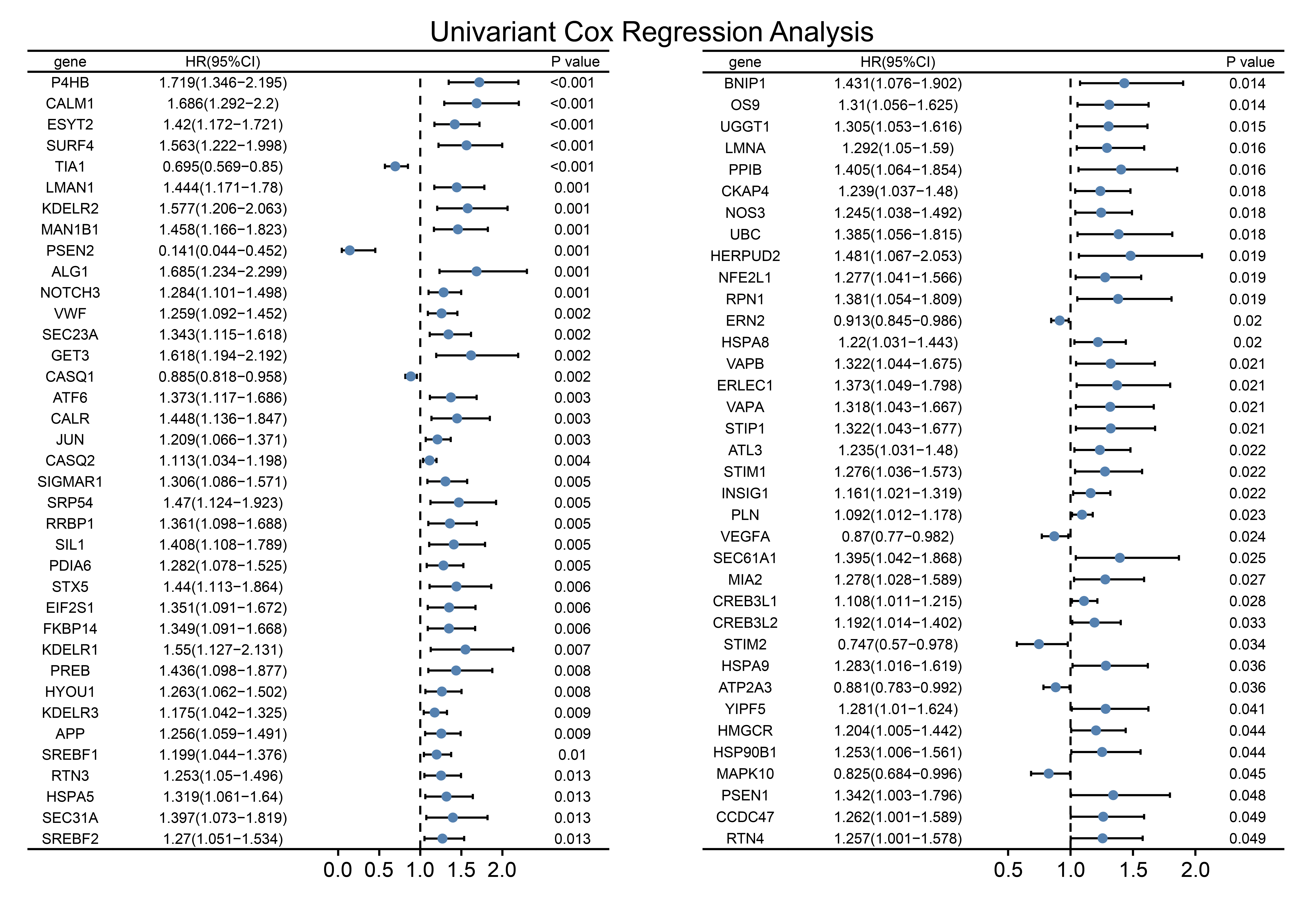

Supplement: Supplementary file 9 [file Image5.JPEG]

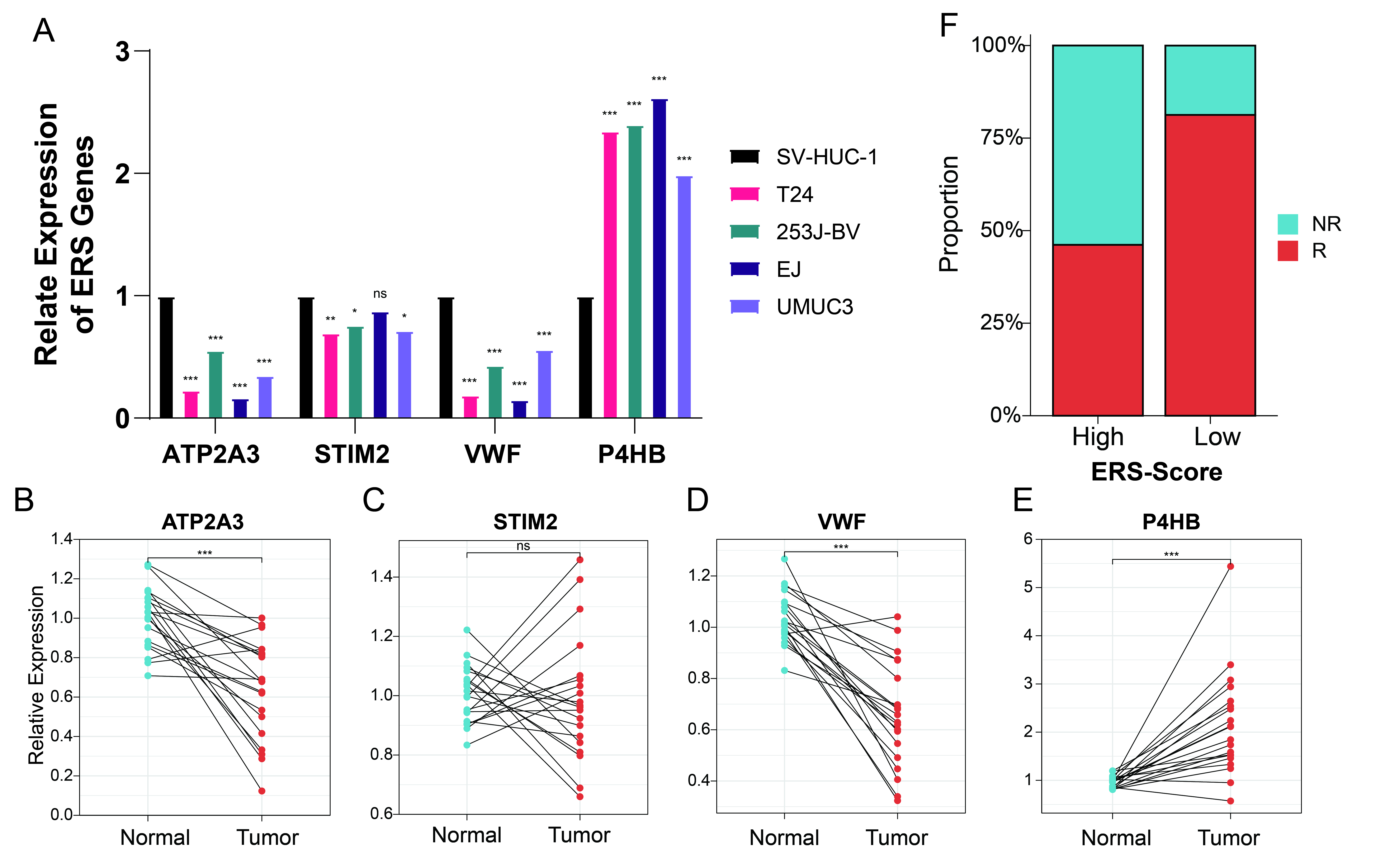

Supplement: Supplementary file 11 [file Image10.JPEG]

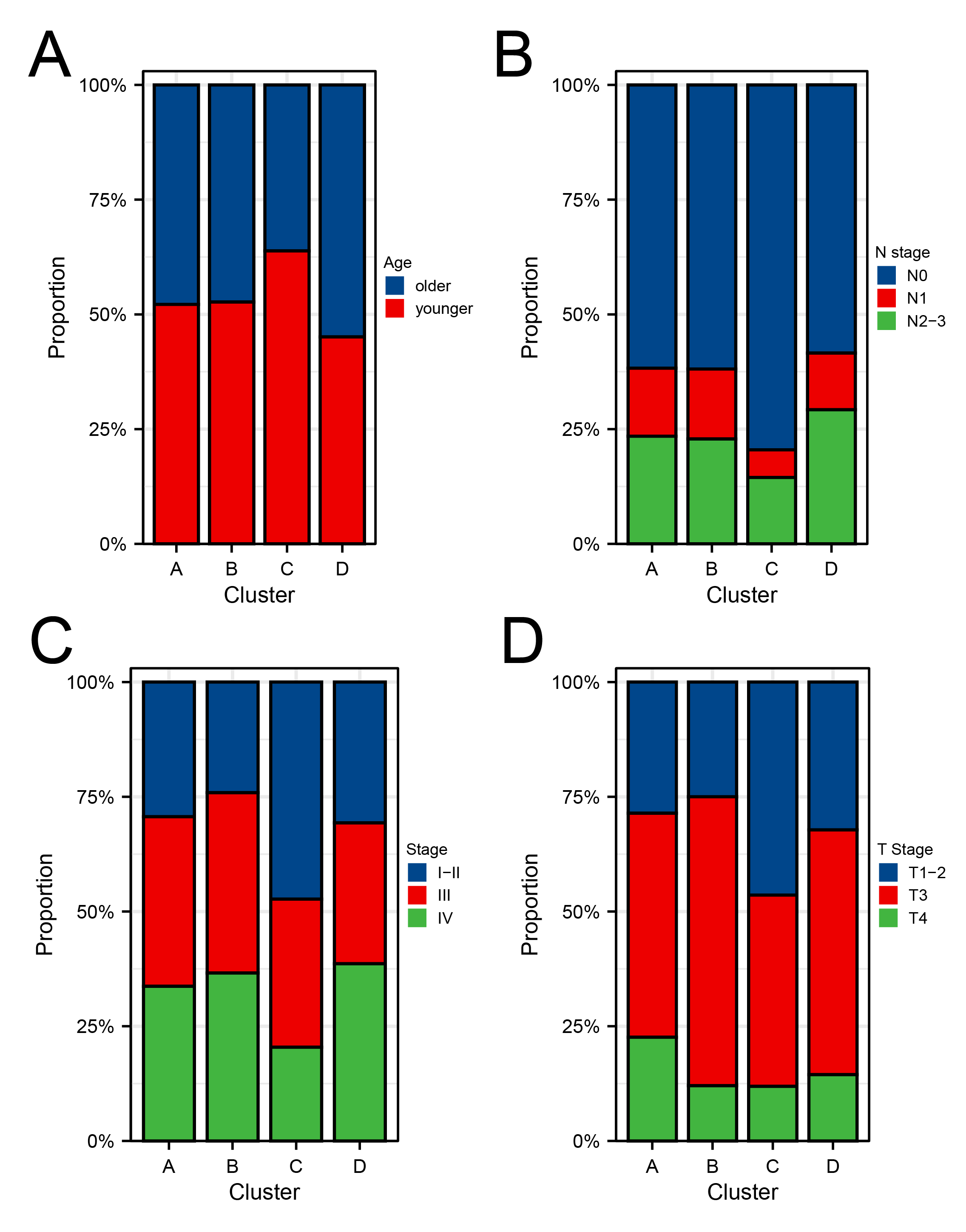

Supplement: Supplementary file 12 [file Image1.TIF]

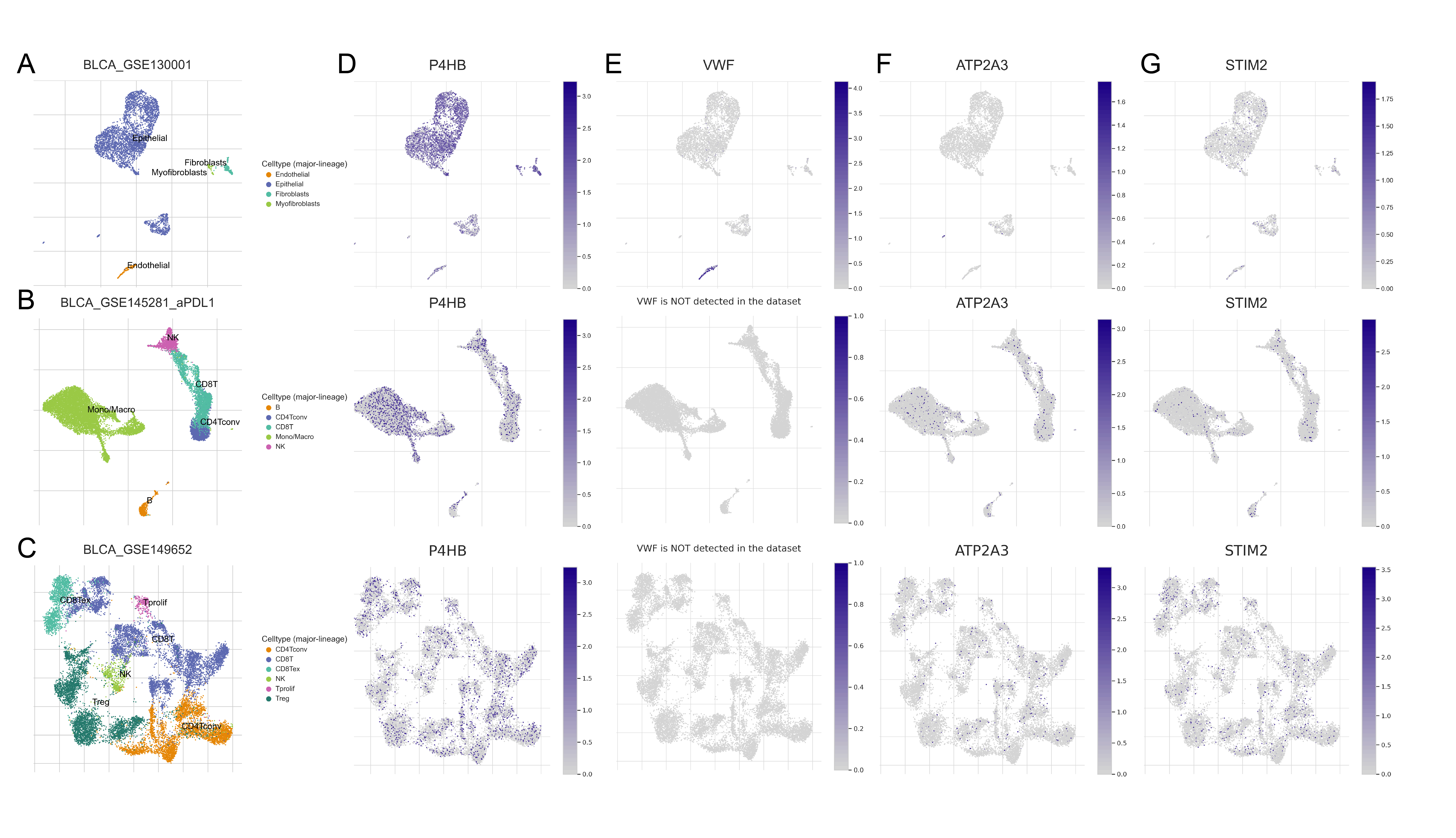

Supplement: Supplementary file 15 [file Image8.JPEG]

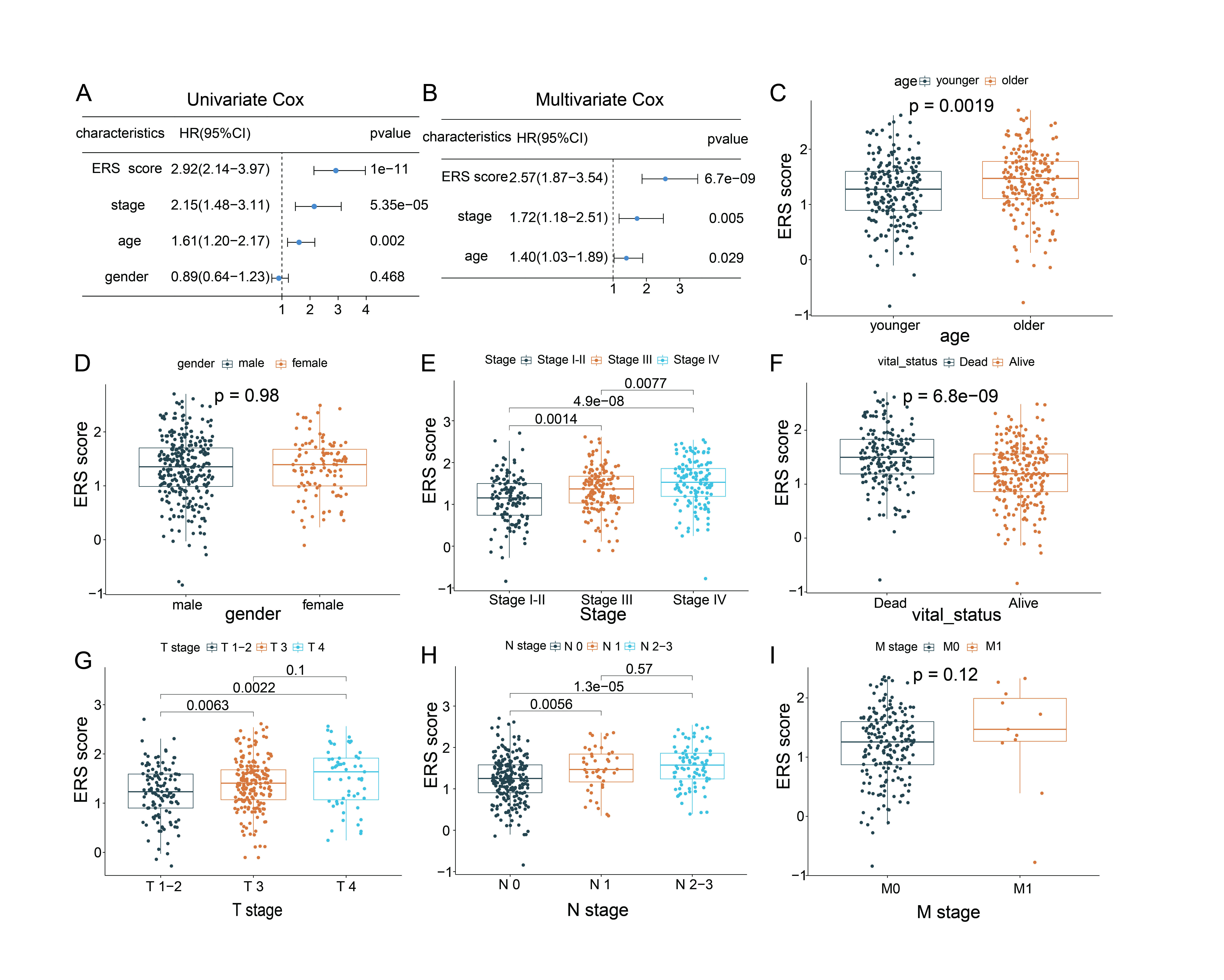

Supplement: Supplementary file 18 [file Image6.JPEG]
